# TRIM56 promotes malignant progression of glioblastoma by stabilizing cIAP1 protein

**DOI:** 10.1186/s13046-022-02534-8

**Published:** 2022-12-06

**Authors:** Xu Yang, Yan Zhang, Zhiwei Xue, Yaotian Hu, Wenjing Zhou, Zhiyi Xue, Xuemeng Liu, Guowei Liu, Wenjie Li, Xiaofei Liu, Xingang Li, Mingzhi Han, Jian Wang

**Affiliations:** 1 Department of Neurosurgery, Qilu Hospital, Cheeloo College of Medicine and Institute of Brain and Brain-Inspired Science, Shandong University, 107 Wenhua Xi Road, 250012 Jinan, Shandong People’s Republic of China; 2 Jinan Microecological Biomedicine Shandong Laboratory, Jinan, 250012 China; 3 Shandong Key Laboratory of Brain Function Remodeling, Jinan, 250012 China; 4 Department of Blood Transfusion, Shandong Provincial Hospital Affiliated to Shandong First Medical University, Jinan, 250022 China; 5 Medical Integration and Practice Center, Cheeloo College of Medicine, Shandong University, Jinan, 250012 China; 6 Department of Biomedicine, University of Bergen, Jonas Lies Vei 91, 5009 Bergen, Norway

**Keywords:** Glioma, Deubiquitylation, TRIM56, cIAP1

## Abstract

**Background:**

The tripartite motif (TRIM) family of proteins plays a key role in the developmental growth and therapeutic resistance of many tumors. However, the regulatory mechanisms and biological functions of TRIM proteins in human glioblastoma (GBM) are not yet fully understood. In this study, we focused on TRIM56, which emerged as the most differentially expressed TRIM family member with increased expression in GBM.

**Methods:**

Western blot, real-time quantitative PCR (qRT-PCR), immunofluorescence (IF) and immunohistochemistry (IHC) were used to study the expression levels of TRIM56 and cIAP1 in GBM cell lines. Co-immunoprecipitation (co-IP) was used to explore the specific binding between target proteins and TRIM56. A xenograft animal model was used to verify the tumor promoting effect of TRIM56 on glioma in vivo.

**Results:**

We observed elevated expression of TRIM56 in malignant gliomas and revealed that TRIM56 promoted glioma progression in vitro and in a GBM xenograft model in nude mice. Analysis of the Human Ubiquitin Array and co-IPs showed that cIAP1 is a protein downstream of TRIM56. TRIM56 deubiquitinated cIAP1, mainly through the zinc finger domain (amino acids 21–205) of TRIM56, thereby reducing the degradation of cIAP1 and thus increasing its expression. TRIM56 also showed prognostic significance in overall survival of glioma patients.

**Conclusions:**

TRIM56-regulated post-translational modifications may contribute to glioma development through stabilization of cIAP1. Furthermore, TRIM56 may serve as a novel prognostic indicator and therapeutic molecular target for GBM.

**Supplementary Information:**

The online version contains supplementary material available at 10.1186/s13046-022-02534-8.

## Background

Almost all oncogenes and tumor suppressors are regulated by post-translational modifications. A critical post-translational modification, ubiquitination, is currently the focus in the study of the regulation of eukaryotic signaling in normal and disease states, as the ubiquitin proteasome pathway is the most important known protein degradation pathway of high specificity in all eukaryotic organisms [1–3].

In the ongoing exploration of the ubiquitin system, the TRIM family was discovered as a new family of ubiquitin ligases and defined as a subfamily of the RING type E3 ubiquitin ligase family [4], some of which are involved in the ubiquitination of proteins [5]. TRIM proteins are widely involved in normal cellular processes, including DNA repair and transfer, regulation of transcription, cell proliferation, autophagy and apoptosis [6, 7]. Several studies have shown that many TRIM proteins, such as TRIM27 and TRIM30α, function as key regulators of innate immunity through signaling of cytokines such as IFN and tumor necrosis factor alpha (TNFα) and pattern recognition receptors (PRRs), such as Toll-like receptors (TLR) [8]. Many TRIM proteins are important regulators of autophagy and innate immunity. Immune-related TRIM proteins, such as TRIM8 and TRIM21, play key roles in oncogenesis or carcinogenesis via NF-kB or IFN signaling pathways [9]. Other TRIM proteins, such as TRIM19, undergo chromosomal translocations, some of which are implicated in the regulation of nuclear receptors, including RARα and hormone receptors [10]. Thus, TRIM proteins, such as TRIM29, may be involved in numerous processes, such as transformation, EMT and metastasis associated with the development of cancer [11, 12].

Glioblastoma (GBM), as one of the most aggressive central nervous system (CNS) cancers of glial cell origin, is the most common primary intracranial tumor accounting for 40%-50% of all gliomas. Despite aggressive treatment, including surgical resection combined with chemotherapy and radiotherapy, tumors inevitably recur [13, 14]. The relationship between TRIM family proteins and GBM is largely unclear. Here, we analyzed publicly available datasets to identify the most differentially expressed TRIM family members in human glioma. We found that TRIM56 is the most highly elevated TRIM family member in clinical specimens and associated with higher grade and poorer clinical prognosis in gliomas. In a functional screen using a ubiquitin array, we identify the apoptosis inhibitor cIAP1 as a candidate molecule downstream of TRIM56 and show that TRIM56 inhibits the K48-linked ubiquitination of cIAP1, thus stabilizing the protein. The findings suggest that TRIM56-regulated post-translational modifications may contribute to glioma development through stabilization of cIAP1 and that TRIM56 may serve as a novel prognostic indicator and therapeutic molecular target for GBM. Therefore, inhibition of endogenous expression of TRIM56 may have therapeutic potential for GBM patients.

## Methods

### Cell culture

U251, U118MG and LN229 cell lines were purchased from the American Type Culture Collection. Patient-derived GBM stem-like cells (GSCs) P3, BG5 and BG7 were previously isolated and characterized from GBM surgical specimens. All human cell lines were validated through short tandem repeat analysis (Cell Cook Biotech Co., Ltd; Guangzhou, China). Primary GBM cell lines P3, BG5 and BG7 were kindly provided by Prof. Rolf Bjerkvig (University of Bergen, Bergen, Norway). U118MG and LN229 cell lines were maintained in Dulbecco's modified Eagle's medium (DMEM; Life Technologies/Thermo Fisher Scientific; Waltham, MA, USA) supplemented with 10% fetal bovine serum (Thermo Fisher Scientific). GBM#P3 cells were cultured in serum-free DMEM/F12 medium (Gibco/Thermo Fisher Scientific) supplemented with 2% B27 neuromix (Thermo Fisher Scientific), epidermal growth factor (20 ng/mL; and basic fibroblast growth factor (10 ng/mL; Thermo Fisher Scientific). Normal human astrocytes (NHA) were obtained from Lonza (Walkersville, MD, USA) and cultured in Astrocyte Medium (ScienCell; Carlsbad, CA, USA) supplemented with the Astrocyte Growth Medium Bullet Kit (ScienCell). Cells were incubated in 5% CO2 at 37 °C.

### Transient transfection and lentiviral infection

Transient transfections were performed for siRNAs with 4 µL of Lipofectamine 2000 (Thermo Fisher Scientific) and 5 µL of siRNA, and for plasmids, using a ratio of 1 µg:1 µL of plasmid to transfection reagent per well in 6-well plates. For lentiviral infection, target cells were seeded, the media removed the following day, and the lentivirus dilution added with polybrene at a final concentration of 5 μg/mL. Cells were incubated for 72 h and selected in puromycin (2 μg/mL) for 2 weeks to achieve stable expression in the cell lines. Sequences of siRNAs and shRNAs used are shown in Supplementary Table S3. The plasmids used and the source are shown in Supplementary Table S4.

### Real-time qPCR (qRT-PCR)

Total RNA was extracted from cells with TRIzol reagent (Thermo Fisher Scientific) and reverse transcribed into cDNA with the ReverTra Ace qPCR RT Kit (Toyota Life Sciences; Shanghai, China) according to the manufacturer's protocols. Real-time PCR was performed on cDNA in the real-time PCR detection system LightCycler 480II (Roche; Basel, Switzerland). GAPDH served as an internal control. The sequences of the primers used for PCR are listed in Supplementary Table S5.

### Colony forming assays

Treated cells were seeded into 6-well plates (1 × 10^3^ cells/well) and cultured for 2 weeks. Cells were fixed with 4% paraformaldehyde and stained with crystal violet. Colonies with over 100 cells were counted for analysis.

### Cell cycle assays

Stable or plasmid-transfected P3, LN229 and U118MG cells were enzymatically dissociated and collected (2000 rpm/min for 5 min). The cells were resuspended in PBS and centrifuged again. The cells were then fixed in 70% ethanol (~ 300 μL). The fixed cells were left overnight at 4 °C, harvested at 2000 rpm/min for 5 min, and rinsed with PBS at 2000 rpm/min for 5 min. Cells were stained with propidium iodide (PI, BD Biosciences; San Jose, CA, USA) for 15–20 min and then subjected to cell cycle analysis by flow cytometry (NovoCyte D3000, Agilent Biosciences, USA). In gate P1, the linear relationship was set for FSC-H (10^6^) and SSC-H (10^6^), and gate P2 was used to highlight the diploid and tetraploid cells.

### Immunohistochemistry (IHC)

Primary GBM and xenograft samples were embedded in paraffin and sectioned (4 μm). IHC was performed as previously described [15]. The following primary antibodies were used in this study: anti-TRIM56 antibody (Abcam, Cambridge, MA, USA; 1:200; cat#ab154862), cIAP1 monoclonal antibody (Proteintech; Rosemont, IL, USA; 1:200; cat#66,626–1-Ig) and anti-Ki67 antibody (Abcam, 1:1000; cat#ab15580). Staining was evaluated independently to determine the histological score according to the proportion of positive staining cells and staining intensity.

### Immunofluorescence (IF)

Sections were deparaffinized and treated with antigen retrieval, blocked with bovine or rabbit serum albumin for 30 min, and incubated with anti-TRIM56 antibody (Abcam, cat#ab154862) and cIAP1 monoclonal antibody (Proteintech, cat#66,626–1-Ig) for 2 h at room temperature or overnight at 4 °C. The samples were then incubated with species-appropriate, fluorescently-labeled or horseradish peroxidase (HRP)-labeled secondary antibodies for 45 min at room temperature at a dilution of 1:500. DAPI (G1012; Servicebio) was used to stain nuclei. Sections were scanned in the Pannoramic MIDI II (3Dhistech; Budapest, Hungary) slide scanner to capture the images.

### Western blotting (WB)

Cells were lysed with RIPA lysis buffer (Thermo Fisher Scientific) supplemented with a protease and phosphatase inhibitor cocktail (Sigma-Aldrich; St. Louis, MO, USA), and protein concentration was determined with the BCA Protein Assay Kit (Beyotime, Shanghai, China) according to the manufacturer's instructions. Equal amounts of protein extracts (5–20 μg) were separated on SDS–PAGE followed by electrotransfer onto a PVDF membrane (Merck Millipore; Billerica, MA, USA). The membrane was blocked with skim milk for 1 h and incubated with primary antibodies for 2 h at room temperature or overnight at 4 °C followed by incubation with a horseradish peroxidase (HRP) conjugated secondary antibody (ZSGB-BIO; Beijing, China) for 1 h. Immunoreactivity was detected with ECL (Merck Millipore), and GAPDH was used as a loading control. The following primary antibodies were used: recombinant anti-TRIM56 antibody (Abcam, cat#ab154862), β-actin mouse monoclonal antibody (CST, cat#3700 s; Danvers, MA, USA), cIAP1 polyclonal antibody (Proteintech, cat#10,022–1-AP), cIAP1 monoclonal antibody (Proteintech, cat#66,626–1-Ig), TNFAIP3 monoclonal antibody (Abcam, cat#ab92324), recombinant anti-DDDDK tag antibody (Abcam, cat#ab205606), recombinant anti-ubiquitin (linkage-specific K48) antibody (Abcam, cat#ab140601), recombinant anti-ubiquitin (linkage-specific K63) antibody (Abcam, cat#ab179434), anti-ubiquitin antibody (Abcam, cat#ab134953), cleaved caspase-3 antibody (CST, cat#9661), cleaved caspase-7 antibody (CST, cat#9491), cleaved caspase-8 antibody (CST, cat#9429), and cleaved caspase-9 antibody (CST, cat#9507).

### Cell invasion assays

For Transwell analysis, cells (3 × 10^4^ cells / well) were seeded into the upper chamber of a 12-well plate in medium without FBS, and medium containing 15% FBS was placed in the lower chamber. The cells that had migrated through the membrane were fixed with 4% paraformaldehyde and stained with crystal violet. Images were acquired under brightfield microscopy and the cells were counted. For the 3D tumor sphere invasion assay, cell spheres were embedded in Matrigel (Trevigen; Gaithersburg, MD, USA) and cultured at 37 °C for 72 h. The sphere diameter was considered to be the starting point for quantification.

For 3D tumor sphere invasion assays, GBM#P3 cells (3 × 10^3^/well) with targeted gene knockdown or overexpression were seeded onto low adherence 96-well plates. After sphere formation, invasion gel (50 μ/well; R&D Systems; 3500–096-03; Minneapolis, MN, USA) was added to the wells, and plates were incubated for 72 h. Images were acquired at specific intervals to examine the invasion ability of modified tumor cells.

In vitro GBM brain organoid co-culture, such as the culture of 18-day rat embryonic brain organoids, was also performed to assess GBM cell invasion. GBM cells transfected with GFP were cultured to allow formation of spheres, and then co-cultured with mature brain organoids for 72 h. Images of co-cultures were acquired under confocal microscopy to assess invasion ability (Leica TCS Sp8; Wetzlar, Germany).

### Ubiquitination screen assay

Targets of TRIM56-mediated ubiquitination were determined using an R&D Systems Proteome Profiler Human Ubiquitin Array Kit (cat#ARY027), which includes 49 different proteins. Relative expression levels of ubiquitination of human proteins in samples were determined according to the manufacturer’s protocol. Briefly, the human ubiquitin array nitrocellulose membranes spotted with 49 different antibodies to human ubiquitin target proteins were incubated with prepared cell lysates for 1 h on a rocking platform shaker. After thorough washing to remove unbound proteins, the membrane was incubated with a biotinylated pan-anti-ubiquitin detection antibody cocktail at 4 °C overnight. The next day the membrane was washed thoroughly, followed by addition of streptavidin-HRP. The signal produced at each capture spot corresponding to the relative amount of ubiquitinated protein bound was exposed to autoradiography film and analyzed.

### Co-immunoprecipitation (Co-IP)

Briefly, cells were lysed in IP lysis buffer (Thermo Fisher Scientific) containing a protease inhibitor mixture (1%, Sigma-Aldrich). Total lysates (200 µg; 1 µg/µL) were incubated with primary antibody (5 µL) or IgG (5 µL) overnight at 4 °C with constant gentle shaking, and then incubated with protein A/G magnetic beads (Thermo Fisher Scientific) for 1–2 h at room temperature. Immunoprecipitated complexes were then detected using an immunoblotting assay.

### Cycloheximide (CHX) chase

GBM#P3, LN229 and U118MG cells were infected with lentivirus (Biosune Biotechnology; Shanghai, China) to generate cell populations with knockdown of TRIM56. GBM#P3, LN229 and U118MG cells were transfected with plasmids to overexpress TRIM56 (Biosune Biotechnology). Modified cells were exposed to CHX (25 μg/mL; Apexbio; Houston, TX, USA) to inhibit translation, and cell lysates were prepared at the indicated times. Protein lysates (20 μg) were run on western blot to detect TRIM56 levels.

### Cell number counting

Cells with targeted gene knockdown or overexpression (1 × 10^5^/well) were seeded into 6-well plates. Cells were collected through trypsin digestion and counted every 24 h. Cells were counted in three wells to obtain average counts, and three independent biological replicates were performed for each experiment.

### Ubiquitination assay

To assess the level of intracellular ubiquitination, plasmids (1 μg/well) expressing genes such as Myc-cIAP1, Flag-TRIM56 and HA-Ub were transfected into cells and incubated with 20 μM MG132 (Apexbio) for 6 h prior to cell extraction. Cells were subsequently lysed and extracted for western blot, IPs or Co-IPs, using antibodies such as: anti-K48/63-linked specific polyubiquitin antibodies or other antibodies.

### Animal studies

To eliminate possible bias, five nude mice (male, 4 weeks old; GemPharmatech Co., Ltd; Nanjing, China) were randomly assigned to each group. A human glioma cell line expressing luciferase (3 × 10^5^ cells suspended in 10 μL PBS) was implanted into the frontal lobes of nude mice using a stereotactic device (KDS310, KD Scientific; Holliston, MA, USA). At days 7, 14, 21 and 28 after implantation, tumor growth was examined using bioluminescence imaging (IVIS Spectral In Vivo Imaging System, PerkinElmer; Hopkinton, MA, USA). Animals were euthanized by the cervical dislocation method when they exhibited any signs of persistent discomfort, such as severe hunched posture, reduced activity, apathy, leg dragging, or weight loss of more than 20%. The brains of the mice were perfused, and subsequent analysis was performed on sections with hematoxylin and eosin staining, and IHC/IF.

### Database availability

Expression analysis was performed on data from different grade glioma and normal brain tissue samples in publicly available databases, including TCGA-GBM, GSE108474, TCGA-LGG GBM, CGGA and Rembrandt, with the EdgeR package. Heatmaps were obtained using the Hiplot website and the EdgeR package. Single cell sequencing data were obtained from the website Single Cell Portal.

### RNA sequencing, data processing and bioinformatics analysis

LN229 mRNA sequencing (mRNA-seq) was performed by the LC Bio Corporation (Hangzhou, China). Sequence analysis was performed on the Illumina PE150 model (Illumina; San Diego, CA, USA). The sequencing depth was 2x, and 3 biological replicate samples were analyzed in each group. Differential gene expression was determined based on fold change (FC; |log2(FC)|) of > 1 and a *P*-value of < 0.05. DAVID was used to perform GO analysis and KEGG analysis, and visualization was accomplished using R software. Differential expression and pathway analysis was performed using gene set enrichment analysis (GSEA).

### Statistical analysis

Experiments in this study used for statistical analyses were performed in at least three independent biological replicates and the results are reported as the mean ± the standard error of the mean (SEM). Statistical significance was calculated using unpaired two-tailed Student's t test for direct comparisons and ANOVA for multiple group comparisons. Survival curves were estimated using the Kaplan–Meier method and compared using the log-rank test. Differences were considered statistically significant when the *P*-value was < 0.05. Statistical analyses were performed using GraphPad Prism version 7.00 software for Windows (GraphPad; La Jolla, CA, USA).

### Role of funding source

The study's funders had no role in the study design, data collection, data analysis, data interpretation, or report writing.

## Results

### TRIM56 is elevated in glioma and associated with poor prognosis

**Fig. 1:**
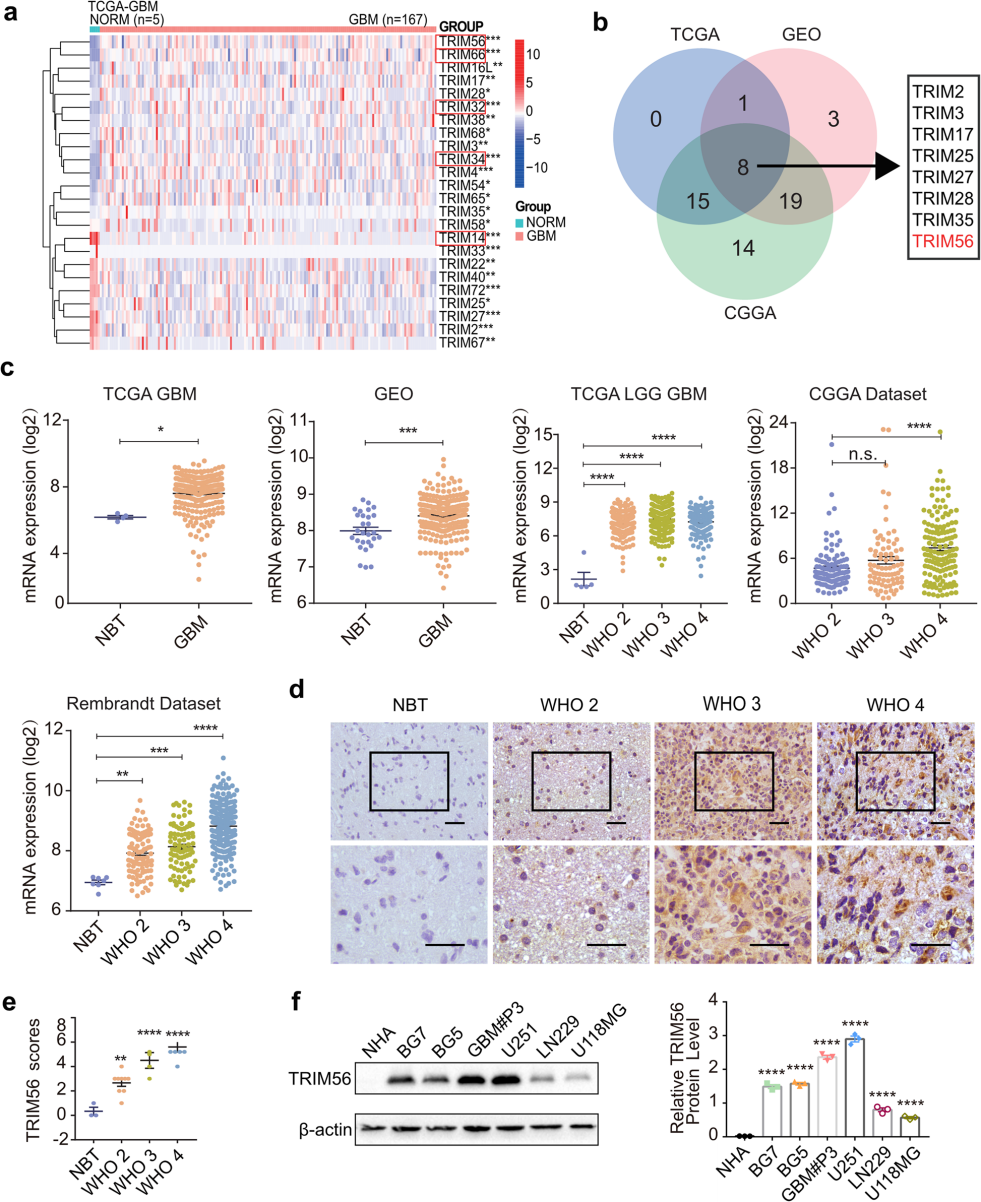
TRIM56 is elevated in glioma and associated with poor prognosis in glioma patients. **a** Heat map of differentially expressed TRIM family genes between normal brain tissue (*n* = 5) and glioblastoma (*n* = 167) from the TCGA-GBM dataset. Gene expression values are z-transformed. The five genes with the most pronounced variability are highlighted by the red boxes. **b** Wayne plots showing eight genes (*TRIM2*, *TRIM3*, *TRIM17*, *TRIM25*, *TRIM27*, *TRIM28*, *TRIM35*, and *TRIM56*) that showed significantly different expression in all three data sets. **c** The expression level of *TRIM56* in glioma patient and normal brain tissues (NBT) in TCGA-GBM, GSE108474, TCGA-LGG GBM, CGGA, and Rembrandt datasets. **d-e** Representative immunostaining and quantification of TRIM56 in different grade human glioma and normal brain tissue (NBT) samples (magnification: × 200, × 400). **f** Western blot and quantification of protein expression levels of TRIM56 in normal human astrocytes (NHAs) and glioma cell lines BG7, BG5, GBM#P3, U251, LN229, and U118MG. Comparisons between two independent samples and among multiple samples were performed using two-tailed t tests and one-way ANOVA, respectively. Error bars indicate at least three independent experiments, and data are shown as mean ± SEM. ∗ *p* < 0.05, ∗  ∗ *p* < 0.01, ∗  ∗  ∗ *p* < 0.001, and ∗  ∗  ∗  ∗ *p* < 0.0001

To determine which member or members of the TRIM family play a pro- or anti-oncogenic role in glioma development and progression, we first investigated the expression levels of TRIM family members in the publicly available TCGA-GBM, GSE108474 and CGGA datasets (Fig. 1a and S1a, b; Supplementary Table S1). To remove the effect caused by tissue specificity, we generated Venn diagrams of TRIM genes with differential expression in these three datasets and showed the number of genes in each overlapping region (Fig. 1b). The Venn diagram yielded eight TRIM genes with significant overexpression in gliomas compared to normal brain tissue from each of the three datasets: *TRIM2*, *TRIM3*, *TRIM17*, *TRIM25*, *TRIM27*, *TRIM28*, *TRIM35*, and *TRIM56*. To further screen for candidate TRIM genes, we performed Kaplan–Meier survival analysis on these eight genes in GEPIA2 (Figure S1c) and found that *TRIM3* and *TRIM56* expression correlated with overall survival in GBM patients. Since *TRIM3* has been shown to have a role as a tumor suppressor in GBM [16–18], we focused our investigation on the function of TRIM56 in the development of GBM.

First, we analyzed the expression levels of *TRIM56* in TCGA-GBM (*n* = 232), GSE108474 (*n* = 256), TCGA-LGG GBM (*n* = 625), CGGA (*n* = 321), and Rembrandt datasets (*n* = 409) (Fig. 1c). We found that *TRIM56* expression was highly upregulated in gliomas compared to normal brain tissue and that increased *TRIM56* expression was positively correlated with increasing grade. Kaplan–Meier survival analysis also showed that LGG and GBM patients with high *TRIM56* expression exhibited poorer overall survival. This result was consistent among multiple datasets (Figure S1d). In addition, we queried the CGGA database for correlation between expression patterns of *TRIM56* and clinical parameters and/or specific molecular subtypes of glioma. We found positive correlation of *TRIM56* expression with glioma grade, IDH wild type, 1p/19q non-coding and age, but no significant correlation with gender and recurrence (Supplementary Figure S2). Univariate and multivariate Cox regression analysis was consistent with the above results, thus verifying the prognostic significance of *TRIM56* in patients with glioma (Supplementary Table S2). We used the study Single cell RNA-seq of adult and pediatric glioblastoma from the Single Cell Portal website to investigate which cell types within the GBM microenvironment express TRIM56 and their expression levels. *TRIM56* is expressed in a variety of cell types, including immune cells, glial cells and tumor cells, and more highly expressed in glioma cells (Supplementary Figure S3). The data together indicated that TRIM56 might function as an oncogene in human gliomas.

To determine whether protein levels were correspondingly increased, we performed immunohistochemistry (IHC) for TRIM56 on an independent cohort of glioma (*n* = 18) and normal brain tissue samples (*n* = 3). TRIM56 expression was low in normal brain tissue, but increased with increasing grade of glioma (Fig. 1d-e). TRIM56 protein levels were also higher in GBM cell lines than in normal human astrocytes (Fig. 1f). In conclusion, the above results suggested that elevated TRIM56 expression was associated with higher grade glioma and may be a potential prognostic marker for GBM patients.

### TRIM56 inhibition inhibits GBM progression in vitro

**Fig. 2:**
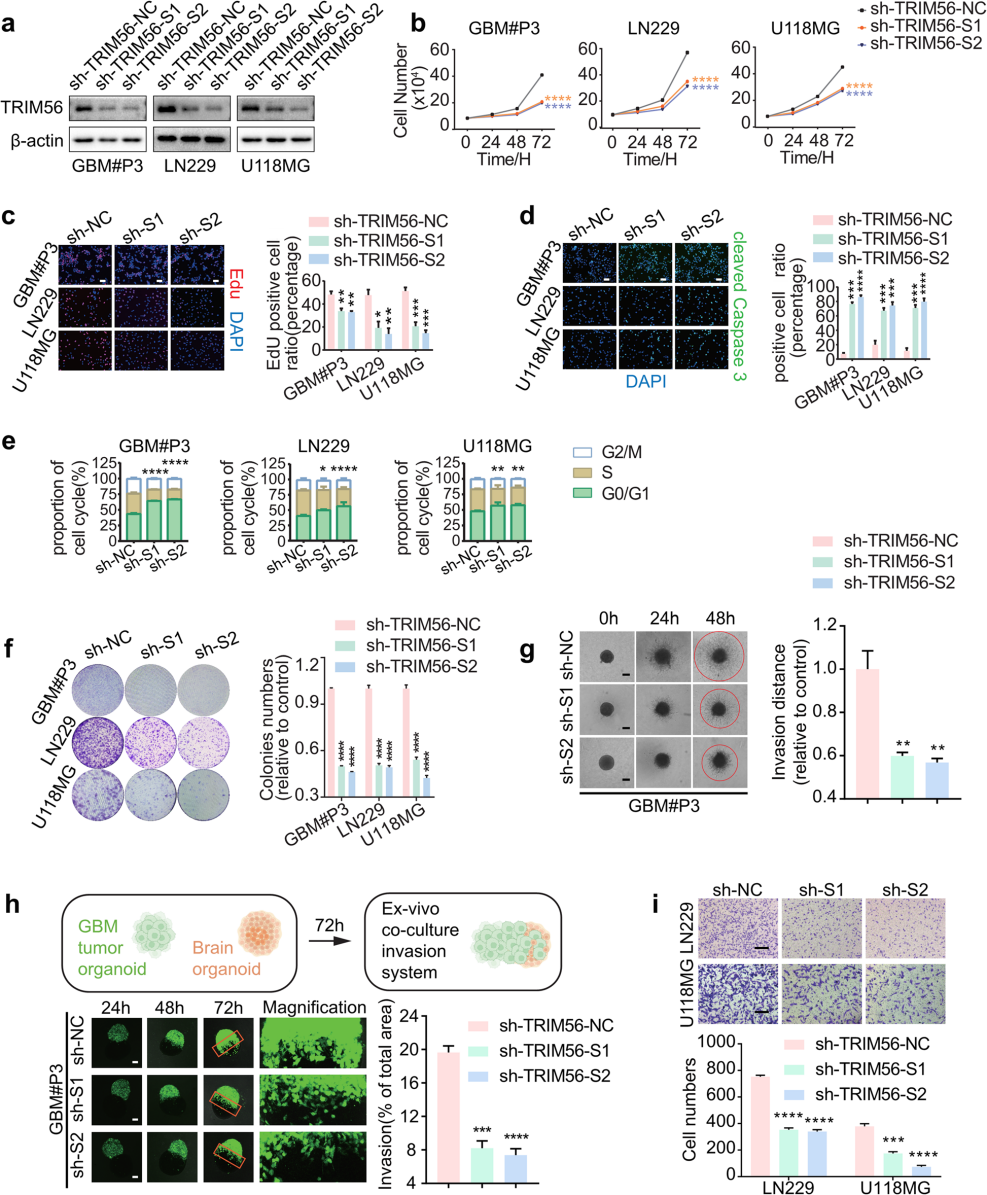
TRIM56 loss inhibits GBM progression in vitro. **a** Western blot showing TRIM56 protein levels in GBM#P3, LN229 and U118MG GBM cell lines stably transduced with lentiviruses containing constructs for shRNAs against TRIM56. **b** Growth curves generated from cell number counts taken over 72 h (at 24-h intervals) to generate growth curves of GBM#P3-, LN229- and U118MG-sh-TRIM56-NC, -S1 and -S2 glioma cells. **c** Fluorescence images and quantification of EdU assays performed to assess the proliferative capacity of GBM#P3-, LN229- and U118MG-sh-TRIM56-NC, -S1 and -S2 glioma cells (scale bar, 500 μm). **d** Fluorescence images and quantification of immunofluorescence staining for cleaved caspase-3 in the indicated cells. Cells were fixed and nuclei were stained with DAPI (scale bar, 500 μm). **e** Cell cycle analysis for the indicated cells. The percentage of cells arrested in the G0/G1 phase is analyzed in bar graphs (right panels). **f** Representative images and quantification of colony formation assays for the indicated cells. Cells were seeded at 1000 cells/well, cultured for 2 weeks, fixed and stained with crystal violet, and quantified. **g** Representative images of 3D tumor sphere invasion assays and quantification for GBM#P3-sh-TRIM56-NC, -S1 and -S2 cells. Representative images were acquired at 0 h, 24 h and 48 h, and plots show percentage of invaded area (scale bar, 200 μm). **h** Model and representative fluorescence images of co-culture invasion assays of GBM#P3-sh-NC, sh-TRIM56-S1 or sh-TRIM56-S2. Invasion capacity was assessed at 24 h, 48 h and 72 h. Scale bars = 200 μm (magnified insets) and the results were quantified. **i** Representative images and quantification of Transwell assays performed on LN229- and U118MG-sh-TRIM56-NC, -S1 and -S2 cells (scale bar, 50 μm). Cells were fixed, stained with crystal violet and counted. Comparisons between two independent samples and among multiple samples were performed using two-tailed t tests and one/two-way ANOVA, respectively. Error bars indicate at least three independent experiments, and data are shown as mean ± SEM. ∗ *p* < 0.05, ∗  ∗ *p* < 0.01, ∗  ∗  ∗ *p* < 0.001, and ∗  ∗  ∗  ∗ *p* < 0.0001

To begin to explore the role of TRIM56 in human glioma, we knocked down expression of TRIM56 in human glioma cell lines GBM#P3, LN229 and U118MG with two independent short hairpin RNAs (shRNA). Cells were infected with lentiviruses expressing sh-TRIM56-S1 or -S2, or the empty vector control, and knockdown efficiency of the shRNAs was confirmed on western blot (Fig. 2a). In both cell number counting and EdU assays, we found that loss of TRIM56 inhibits proliferation of GBM#P3, LN229 and U118MG cells (Fig. 2b-c). In addition, immunofluorescence demonstrated a significant increase in the proportion of cleaved caspase-3-positive cells in TRIM56-deficient GBM#P3, LN229 and U118MG cells, which suggested that loss of TRIM56 caused apoptosis (Fig. 2d). Cell cycle analysis showed that knockdown of TRIM56 led to an increase of cells in G0/G1 (Fig. 2e). The single cell colony forming ability of GBM#P3-, LN229- and U118MG-shTRIM56 cells was also significantly decreased relative to controls (Fig. 2f).

We investigated whether loss of TRIM56 influenced the invasive properties of GBM cells in several assays exploiting the growth of the cell patient-derived GBM#P3 cells in suspension culture and LN229 and U118MG cells in monolayer. First, we examined the invasive properties of GBM#P3-shTRIM56 cells in a 3D sphere invasion assay. The relative invasion of GBM#P3-shTRIM56 spheroids into the Matrigel was reduced compared to the control cell population (Fig. 2g). To more closely mimic the physiologically aggressive tumor microenvironment, we next introduced GBM#P3-shTRIM56 cells into an ex vivo co-culture invasion model of tumor spheroids with normal rat brain-like organoids, as previously described [19]. In this ex vivo model, invasion of P3-sh-TRIM56 tumor spheroids into rat brain-like organoids was reduced relative to the control cell population (Fig. 2h). We then investigated the invasive ability of modified LN229 and U118MG cells in the Transwell migration assay, and found that the number of cells crossing the cell membrane was significantly reduced for both cell lines compared to controls (Fig. 2i).

In contrast, overexpression of TRIM56 enhanced proliferation of GBM#P3, LN229 and U118MG cells (Figure S4a), as assessed in cell number counting and EdU assays (Figure S4b-c). The proportion of cleaved caspase-3 positive GBM#P3-, LN229- and U118MG-TRIM56OE cells was also reduced (Figure S4d). Overexpression of TRIM56 decreased the number of cells in G0/G1 in glioma (Figure S4e) and enhanced the colony forming ability relative to controls (Figure S4f). Finally, invasion and migration of GBM#P3-TRIM56^OE^ cells was increased relative to controls in the tumor sphere invasion assays (*P* = 0.0321; Figure S4g) and co-culture assays (*P* = 0.0098; Figure S4h). Similarly, Transwell migration of LN229- and U118MG-TRIM56 ^OE^ cells was enhanced relative to control cells (Figure S4i).

In summary, while knockdown of TRIM56 suppressed malignant behavior, overexpression of TRIM56 promoted proliferation and migration of glioma cells in vitro.

### TRIM56 promotes malignant progression of glioma in vivo

**Fig. 3:**
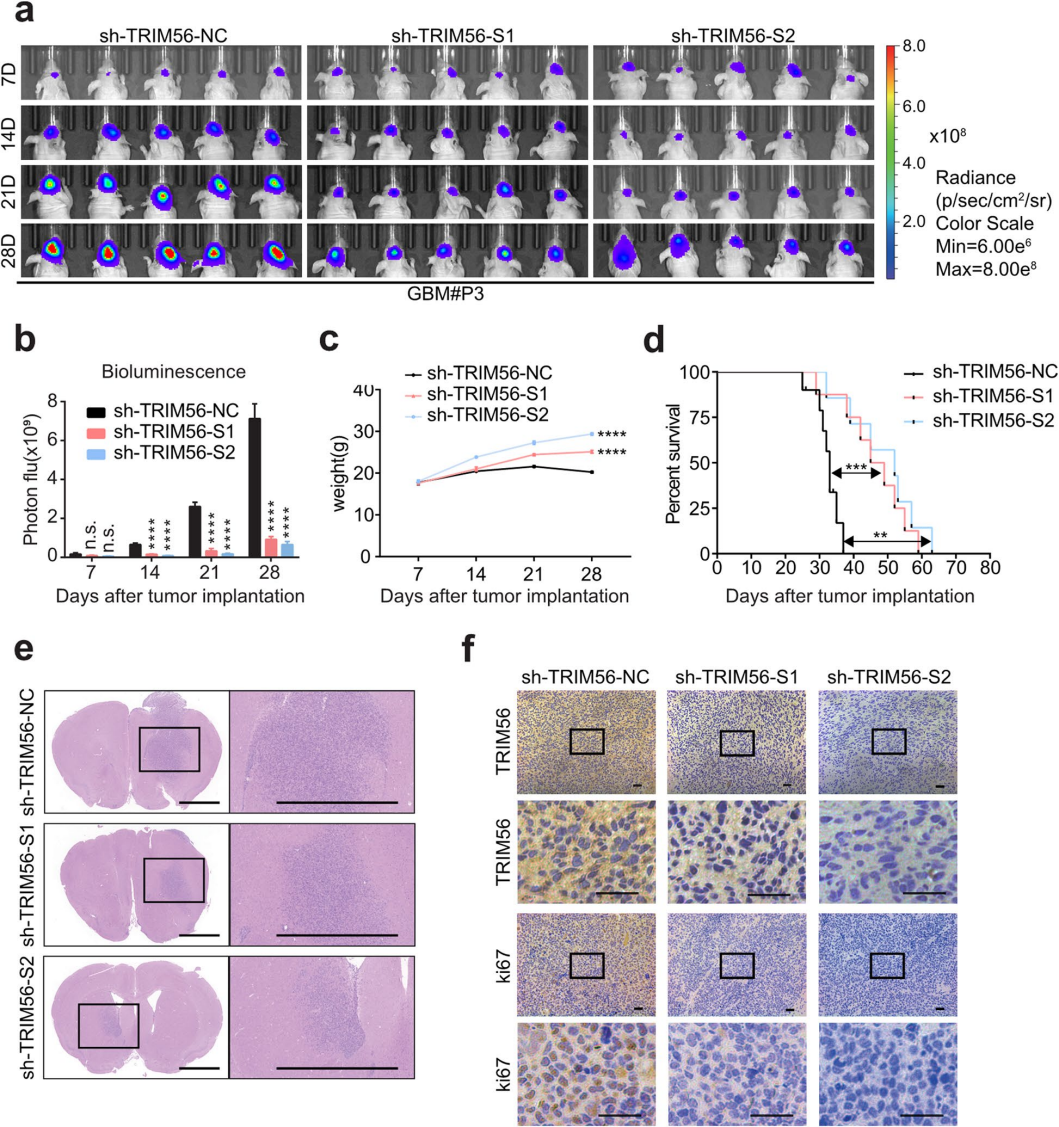
TRIM56 promotes malignant progression of glioma in vivo. **a-b** In vivo bioluminescence imaging and quantification of xenografts derived from lentivirus-infected GBM#P3 sh-TRIM56-NC, -S1, and -S2 cells at the indicated time points (data from 5 animals/group). Representative bioluminescence images at day 7, day 14, day 21 and day 28 post-implantation are shown. Two-way ANOVA and log rank analysis were used to determine the statistical significance of bioluminescence on day 28 (P1 < 0.0001; P2 < 0.0001). Data are shown as the mean ± SEM. **c-d** Weight and Kaplan–Meier analysis of survival time for nude mice implanted with the GBM#P3 cells performed at the indicated time points. Data are shown as the mean ± SEM of three independent experiments. Two-way ANOVA and log-rank analysis were used to determine statistical significance (scale bar, 100 µm). **e** Representative images of HE staining of brain sections from xenografted mice on the day of euthanasia, showing representative macroscopic features. Scale bar = 2 mm. **f** Representative images of immunostaining of TRIM56 and Ki67 in xenograft sections (scale bar, 100 µm). Comparisons between two independent samples and among multiple samples were performed using two-tailed t tests and one/two-way ANOVA, respectively. Error bars indicate at least three independent experiments, and data are shown as mean ± SEM. ∗ *p* < 0.05, ∗  ∗ *p* < 0.01, ∗  ∗  ∗ *p* < 0.001, and ∗  ∗  ∗  ∗ *p *< 0.0001

To determine whether TRIM56 promotes GBM cell growth in vivo, we constructed xenograft mouse models through orthotopic implantation of GBM#P3- and LN229-shTRIM56-1 and -2 cells. Tumor size was significantly reduced for GBM#P3- and LN229-sh-TRIM56 xenografts relative to controls on the 28th day after implantation (GBM#P3, P1 < 0.0001, P2 < 0.0001; LN229, P1 < 0.01, P2 < 0.001; Fig. 3a-b and S5a-b), while the body weight of the mice was increased to some extent relative to control animals (GBM#P3, P1 < 0.0001, P2 < 0.0001; LN229, P1 < 0.0001, P2 < 0.0001; Fig. 3c and S5c). Kaplan–Meier analysis furthermore revealed that the survival of animals with GBM#P3-shTRIM56 tumors was prolonged relative to controls (median survival: 33 versus 49 days for GBM#P3; 34 versus 52 days for LN229; Fig. 3d and S5d). HE (Fig. 3e and S5e) and Ki67 staining (Fig. 3f and S5f) demonstrated that loss of TRIM56 reduced proliferation in tumors. Overall, the results further demonstrated a role for TRIM56 as an oncogene in the development of human glioma.

### RNA-seq analysis reveals potential biological signaling and functions regulated by TRIM56 in glioma

To further investigate the function of TRIM56 in glioma development, we performed RNA-seq analysis on LN229-sh-TRIM56 and LN229 control cells. Each group included three biological replicates. Scatter plots, volcano plots and heat maps revealed 1003 upregulated genes and 539 downregulated genes in LN229-sh-TRIM56 cells relative to controls. (Figure S6a-c). GO analysis and KEGG analysis indicated that these differentially expressed genes were enriched in terms such as protein binding and pathways in cancer (Figure S6d, e). Many of the top 20 enriched pathways identified through KEGG analysis are associated with glioma progression (Figure S6f).

To identify pathways that may be altered by TRIM56 knockdown, we performed GSEA on the RNA-seq data. Representative results showed that signal regulation protein family interactions and apoptosis were closely associated with TRIM56 expression (Figure S6g-i). This result is consistent with previous findings that TRIM56 promotes glioma development by regulating apoptosis.

### cIAP1 is a downstream protein molecule of TRIM56 in glioma

**Fig. 4:**
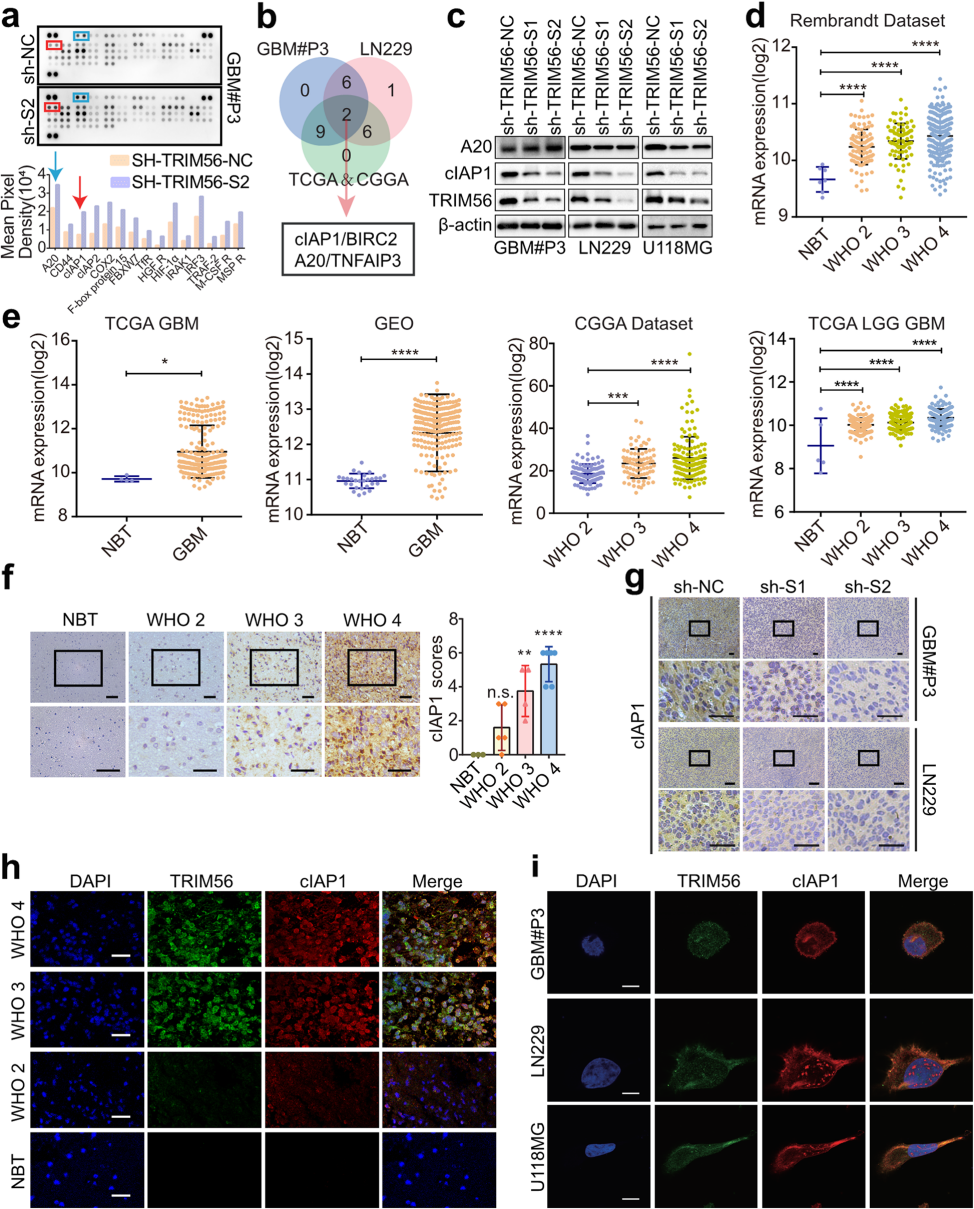
cIAP1 is a downstream protein molecule of TRIM56 in glioma. **a** Images of Human Ubiquitin Array (500 μg lysate, 2 min exposure) performed with lysates prepared from GBM#P3-NC and -sh-TRIM56 cells treated with 20 μg/mL of the proteasome inhibitor MG132 for 6 h before collection. Specific quantification of spots with significantly elevated levels of ubiquitination was performed and the two candidate spots are highlighted in the colored boxes. **b** Venn diagrams showing the intersection of genes that are differentially expressed in both the TCGA and CGGA databases and also show changes in the ubiquitin arrays for both GBM#P3 and LN229 cells in a. **c** Western blot analysis of two candidate target proteins, cIAP1 and A20, in GBM#P3-, LN229- and U118MG-shTRIM56-NC, -S1 and -S2 cells. **d-e** Expression levels of *cIAP1* in glioma patient tissue and normal brain tissue based on TCGA-GBM, GSE108474, TCGA-LGG GBM, CGGA and Rembrandt datasets. **f** Representative images and quantification of immunostaining of cIAP1 in different grades of human glioma and normal brain tissue (NBT) samples (magnification: × 200, × 400). **g** Representative images of immunostaining for cIAP1 in sections from intracranial xenografts derived from GBM#P3- and LN229-shTRIM56-NC, -S1 and -S2 implanted in nude mice (scale bar, 100 µm). **h** Fluorescence images of immunofluorescence staining for TRIM56 and cIAP1 in different grades of human glioma and normal brain tissue samples (NBT) acquired using confocal microscopy (scale bar, 5 µm). **i** Fluorescence images of immunofluorescence staining for TRIM56 and cIAP1 proteins to determine subcellular localization in GBM#P3, LN229 and U118MG glioma cell lines. Immunofluorescence analysis was performed using specific antibodies as shown and images were acquired using confocal microscopy (scale bar, 40 µm). DAPI was used for staining nuclei. Comparisons between two independent samples and among multiple samples were performed using two-tailed t tests and one-way ANOVA, respectively. Error bars indicate at least three independent experiments, and data are shown as mean ± SEM. ∗ *p* < 0.05, ∗  ∗ *p* < 0.01, ∗  ∗  ∗ *p* < 0.001, and ∗  ∗  ∗  ∗ *p* < 0.0001

As a member of the TRIM family, TRIM56 is most likely involved in the ubiquitination or de-ubiquitination of other proteins [20]. Therefore, we screened for candidate proteins ubiquinated by TRIM56 in GBM#P3 cells and LN229 cells, using the Human Ubiquitin Array. We found that the ubiquitination of several proteins on the array was elevated in cells with knockdown of TRIM56 relative to the controls (Fig. 4a and S7a). To eliminate effects arising from cell specificity, we looked for genes in the intersection of differentially expressed genes in the TCGA and the CGGA databases and proteins showing changes in the ubiquitin arrays for both GBM#P3- and LN229-sh-TRIM56-S2 cells (Fig. 4b). The Venn diagram revealed 2 candidate proteins, namely apoptosis inhibitor 1(cIAP1)/BIRC2 and A20/Tumor necrosis factor, alpha-induced protein 3 (TNFAIP3).

To explore whether cIAP1 or A20 is downstream of TRIM56, we first examined protein levels in GBM#P3, LN229 and U118MG cells. We found that cIAP1 and TRIM56 were both highly expressed in glioma cells, and that the expression of cIAP1, but not A20, was significantly decreased with knockdown of TRIM56 in GBM#P3-, LN229- and U118MG-sh-TRIM56-S1 and -S2 cells (Fig. 4c). Thus, we investigated cIAP1 as a potential downstream target of TRIM56.

cIAP1 belongs to the family of inhibitors of apoptosis (IAPs) and has a variety of biological activities, including binding and inhibition of cystathionin, regulation of cell cycle progression, and modulation of receptor-mediated signal transduction [21, 22]. Several studies have already demonstrated that cIAP1 is highly expressed in a variety of human cancers and plays a pivotal carcinogenic role [23, 24]. However, few studies have examined the role of cIAP1 in the development of glioma. Analysis of the TCGA-GBM, TCGA-LGG GBM, CGGA, and GSE108474 datasets revealed that *cIAP1* is highly expressed in gliomas relative to normal brain tissue, and glioma patients with high *cIAP1* tumors have worse survival (Fig. 4d-e and S7b). Since TRIM56 knockdown significantly downregulated cIAP1 expression and cIAP1 has been documented to play an oncogenic role in a variety of cancers, we hypothesized that cIAP1 may be a key molecule mediating the malignant function of TRIM56 in gliomas. Immunohistochemical staining of tumor samples demonstrated that cIAP1 protein was highly expressed in gliomas, and that expression was increased in high-grade gliomas (Fig. 4f). In contrast, cIAP1 protein levels were decreased in tumor xenografts derived from GBM#P3- and LN229-shTRIM56-1 and -2 cells relative to controls (Fig. 4g).

Finally, images of immunofluorescence staining acquired with confocal microscopy showed that TRIM56 co-localized with cIAP1 in human gliomas, normal brain tissue samples and GBM cells (Fig. 4h-i).

In conclusion, these results suggested that cIAP1 is a potential substrate for TRIM56, involved in the malignant proliferation and invasion of glioma cells.

### TRIM56 promotes the malignant development of glioma by enhancing the stability of cIAP1

**Fig. 5:**
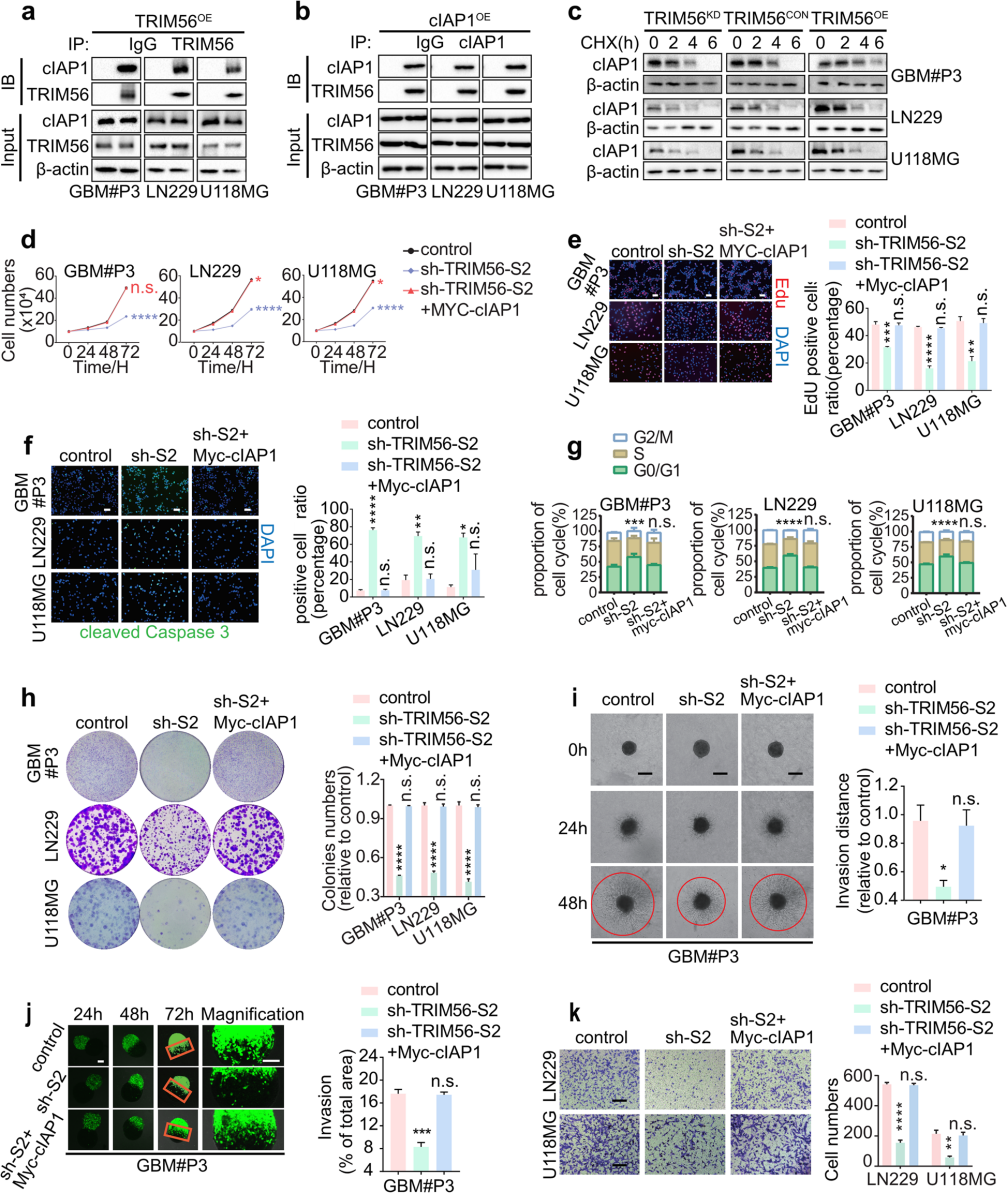
TRIM56 promotes the development of glioma by enhancing the stability of cIAP1. **a-b** GBM#P3-, LN229- and U118MG-TRIM56^OE^ cells were immunoprecipitated using IgG antibody or TRIM56/ cIAP1 antibody and were immunoblotted using TRIM56 and cIAP1 antibodies. **c** Western blot of lysates prepared from GBM#P3-, LN229- and U118MG-TRIM56^KD^, -TRIM56^CON^ and -TRIM56.^OE^ cells treated with 25 μg/mL of cycloheximide (CHX) for the indicated times to determine half-life of cIAP1 protein. Blots are incubated with the antibodies indicated **d** Growth curves generated from cell number counts performed on the cells over a 72-h period (at 24-h intervals). **e** Fluorescence images and quantification of EdU assays performed on cells (scale bar, 500 μm). **f** Fluorescence images and quantification of staining for cleaved caspase-3. DAPI was used to stain nuclei (scale bar, 500 μm). **g** Cell cycle analysis for the indicated cells. The percentage of cells arrested in the G0/G1 phase is analyzed in the bar graphs below. **h** Representative images of colony formation assays and quantification on cells shown (GBM#P3- LN229- and U118MG-shTRIM56-NC, -shTRIM56-S2 and -shTRIM56-S2 cells transfected with Myc-cIAP1) seeded at 1000 cells/well and cultured for 2 weeks. **i** Brightfield images of 3D tumor sphere invasion assays and quantification for GBM#P3 cells transfected with plasmid for Myc-cIAP1 or GBM#P3-shTRIM56-S2 cells. Representative images at 0 h, 24 h and 48 h are shown, as well as quantitative plots of the percentage of invaded area (scale bar, 200 μm). **j** Representative images of co-culture invasion assays and quantification for GBM#P3 cells. Invasion was assessed at 24 h, 48 h and 72 h (scale bars = 200 μm). **k** Representative images of Transwell assays and quantification performed on LN229- and U118MG-shTRIM56-S2 cells. Cells were fixed, stained with crystal violet and counted (scale bar, 50 μm). Comparisons between two independent samples and among multiple samples were performed using two-tailed t tests and one/two-way ANOVA, respectively. Error bars indicate at least three independent experiments, and data are shown as mean ± SEM. ∗ p < 0.05, ∗  ∗ *p* < 0.01, ∗  ∗  ∗ *p* < 0.001, and ∗  ∗  ∗  ∗ *p* < 0.0001

We next investigated the mechanisms of TRIM56 regulation of cIAP1 protein levels. To examine *cIAP1* mRNA levels in response to TRIM56, we performed qRT-PCR on RNA from GBM#P3-, LN229- and U118MG-sh-TRIM56-S1 and -S2 cells and found that *cIAP1* mRNA levels remained unaltered with the knockdown of TRIM56 (Figure S7c). These results suggested that TRIM56 did not regulate cIAP1 expression at the RNA level. We then overexpressed TRIM56 and cIAP1 in GBM#P3, LN229 and U118MG cells, and performed Co-IPs to determine whether the proteins were in complexes together. Complexes containing both TRIM56 and cIAP1 were detected in all three cell populations overexpressing the two proteins (Fig. 5a-b). More importantly, complexes containing TRIM56 and cIAP1 were also detected in the parental cell lines (Figure S7d-e). We also found that transfection of TRIM56 into GBM#P3-, LN229- and U118MG-cIAP1^OE^ cells further increased cIAP1 protein levels (Fig. 5c). These results indicated that TRIM56 enhanced the expression of cIAP1 at the protein level.

We next treated cells with cycloheximide to examine the half-life of cIAP1 in cells with TRIM56 overexpression (OE) or TRIM56 knockdown (KD) (Fig. 5c). The levels and half-life of cIAP1 protein were increased in GBM#P3-, LN229- and U118MG-TRIM56^OE^ compared to control groups. In contrast, the half-life of cIAP1 was decreased in GBM#P3-, LN229- and U118MG-TRIM56^KD^. Therefore, TRIM56 stabilized cIAP1 protein through post-translational modification in GBM cell lines.

We also examined whether cIAP1 restored clonogenic, proliferative and invasive properties of cells with loss of TRIM56. Overexpression of cIAP1 (Myc-cIAP1) increased cell number and percentage of EdU positive cells of GBM#P3-, LN229- and U118MG-sh-TRIM56-S2 cells (Fig. 5d-e) which demonstrated that overexpression of cIAP1 restored the proliferative capacity of GBM cells with loss of TRIM56. Immunofluorescence staining for cleaved caspase-3 demonstrated that overexpression of cIAP1 decreased apoptosis in GBM#P3-, LN229- and U118MG-shTRIM56-S2 cells and thus rescued cells from TRIM56 knockdown (Fig. 5f). cIAP1 rescued the cell cycle parameters (Fig. 5g) and the clonogenic ability (Fig. 5h) of glioma cells with TRIM56 knocked down.

We next asked whether cIAP1 restored invasive properties of GBM cells with TRIM56 knockdown. GBM#P3-shTRIM56-S2 cells showed increased invasion in the 3D invasion sphere (Fig. 5i) and the ex vivo co-culture invasion model (Fig. 5j) with overexpression of Myc-cIAP1. An increased number of LN229- and U118MG-sh-TRIM56-S2 cells migrated across the membrane in Transwell migration assays of (Fig. 5k) with overexpression of cIAP1. Thus, cIAP1 rescued the invasive ability of glioma cells that had been suppressed due to TRIM56 knockdown.

We then examined the expression of cIAP1-related genes, including cleaved caspase-3, cleaved caspase-7, and cleaved caspase-9 [25]. Western blot analysis demonstrated that levels of cleaved caspase-3, cleaved caspase-7, and cleaved caspase-9 were increased in GBM#P3-, LN229- and U118MG-sh-TRIM56-S1 and -S2 cells relative to controls (Figure S7f). These results further indicated that TRIM56 promoted the development of glioma through cIAP1.

Taken together, we demonstrated that TRIM56 promoted the malignant behavior of glioma cells by enhancing the stability of cIAP1 protein.

### TRIM56 stabilizes cIAP1 via deubiquitylation

**Fig. 6:**
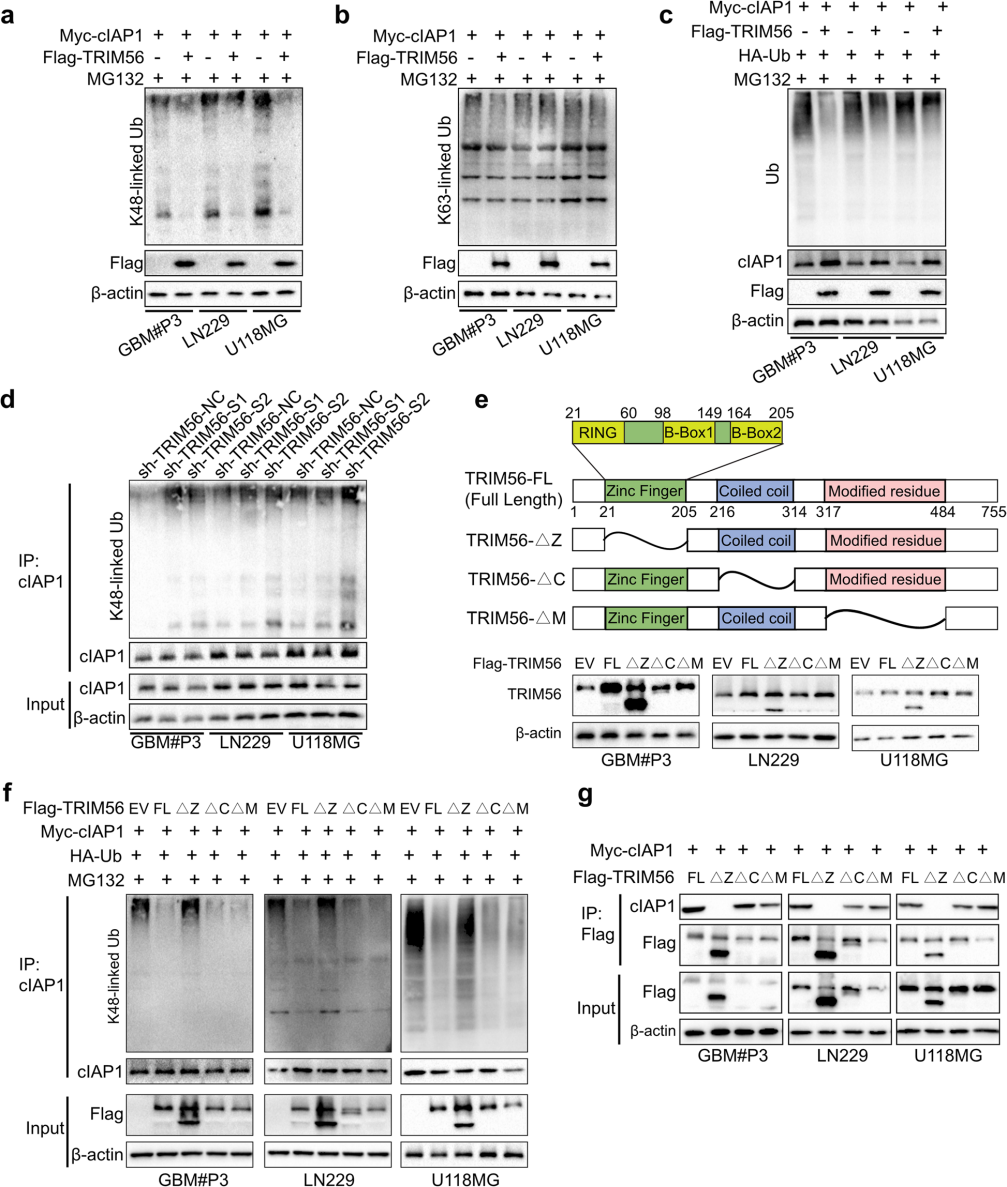
TRIM56 stabilizes cIAP1 via deubiquitylation. **a-b** Western blot of lysates prepared from GBM#P3-, LN229- and U118MG-cIAP1^OE^ cells transfected with Flag-TRIM56 and treated with MG132 (20 μg/mL) for 6 h before collection. Immunoblotting was then performed using K48/63-linked Ub and Flag antibodies. **c** Western blot analysis performed on extracts prepared from GBM#P3, LN229 and U118MG cells transfected with Myc-cIAP1, Flag-TRIM56 and HA-Ub (as indicated) and treated with MG132 (20 μg/mL) for 6 h prior to collection with indicated antibodies. **d** Western blot of co-IPs performed on extracts prepared from the stably transfected GBM#P3, LN229 and U118MG cells shown and pulled down with cIAP1 antibody. Blots were incubated with the antibodies indicated including K48-linked specific polyubiquitin antibody. **e** Schematic representation of the functional domains contained in wild-type TRIM56 and each deletion mutant and western blot of lysates prepared from GBM#P3 cells, LN229 cells and U118MG cells transfected with the different TRIM56 full length and mutant plasmids. Blots were incubated with the antibodies shown. **f** Western blot of co-IPs performed on extracts prepared from GBM#P3, LN229 and U118MG cells transfected with Myc-cIAP1, HA-Ub and full length or deletion mutants of Flag-TRIM56 as indicated and treated with MG132 (20 μg/mL) for 6 h prior to collection. cIAP1 antibody was used in the immunoprecipitations. **g** Western blot of co-IPs performed on lysates prepared from cells transfected with Myc-cIAP1 and full length or deletion mutants of Flag-TRIM56 in GBM#P3, LN229 and U118MG cells to determine the interaction between TRIM56 structural domains and cIAP1. Cell lysates were immunoprecipitated with anti-Flag. Comparisons between two independent samples and among multiple samples were performed using two-tailed t tests and one-way ANOVA, respectively. Error bars indicate at least three independent experiments, and data are shown as mean ± SEM. ∗ *p *< 0.05, ∗  ∗ *p* < 0.01, ∗  ∗  ∗ *p* < 0.001, and ∗  ∗  ∗  ∗ *p* < 0.0001

We next investigated whether TRIM56 interacts directly with cIAP1 and stabilizes the protein through a deubiquitinating enzyme. We therefore first examined the effect of TRIM56 on proteasome-associated poly-ubiquitination, such as K48- or K63-linked ubiquitin. We transfected GBM#P3-, LN229- and U118MG-cIAP1^OE^ cells with Flag-TRIM56, treated cells with the proteasome inhibitor MG132 (20 μg/mL; treated for 6 h before collection) and then detected K48/63-linked ubiquitin on western blot. We found that overexpression of TRIM56 was associated with reduced total K48-linked polyubiquitination levels in all cell lines (Fig. 6a). However, K63-linked ubiquitination levels were not significantly changed (Fig. 6b).

We next tested whether TRIM56 reduced ubiquitination levels in cIAP1. We transfected cIAP1 (Myc-cIAP1), ubiquitin (HA-Ub) and TRIM56 into GBM cell lines, and treated them with MG132. Immunoblot analysis of glioma cell lines transfected with TRIM56 showed reduced levels of polyubiquitinated cIAP1 (Fig. 6c). To examine whether TRIM56 targeted K48-linked ubiquitination of cIAP1, IPs were performed to pull down cIAP1 in the GBM#P3-, LN229- and U118MG-sh-TRIM56-S1 and -S2 cells, and K48-linked ubiquitin was then detected with antibodies on western blot. Increased levels of K48-linked ubiquitin were detected in cIAP1 pulled down from all TRIM56 knockdown cell populations relative to controls (Fig. 6d). Taken together, the results suggested that TRIM56 enhanced the stability of cIAP1 protein by inhibiting K48-linked polyubiquitination of cIAP1.

To identify the structural domain of TRIM56 interacting directly with cIAP1 and/or causing deubiquitylation, we constructed the following three mutants: zinc finger domain, amino acids 21–205 (△Z); coiled coil domain, amino acids 216–314 (△C); and modified residue, amino acids 317–484 (△M) (Fig. 6e). The zinc finger domain includes a RING domain (amino acids 21–60) and two B-Box domains (amino acids 98–149 and amino acids 164–205). We transfected Myc-cIAP1, HA-Ub and the TRIM56 mutants into GBM#P3, LN229 and U118MG cells and treated them with MG132. IPs were performed on extracts with cIAP1 antibody and western blots were probed with K48-linked ubiquitin antibody. The TRIM56 mutant containing the complete zinc finger domain (△C and △M) inhibited K48 polyubiquitination similar to TRIM56-FL, whereas the mutant missing the zinc finger domain (△Z) failed to inhibit K48 polyubiquitination (Fig. 6f). Compared with other deleted structural domains, the zinc finger structural domain reduced the polyubiquitination of cIAP1, indicating that the zinc finger structural domain is essential for the deubiquitinating of cIAP1.

To investigate which functional domains of TRIM56 interact with cIAP1, we transfected cIAP1 with various deletion mutants of TRIM56 into glioma cell lines and performed co-IPs. Only the △Z mutant failed to pull down cIAP1 (Fig. 6g), which demonstrated that cIAP1 interacts with the zinc finger domain of TRIM56.

Taken together, the data from this assay support that TRIM56 interacts with cIAP1 via the functional region between amino acids 21–205 (zinc finger domain), thereby inhibiting K48-linked polyubiquitination of cIAP1, reducing cIAP1 degradation and promoting glioma development.

## Discussion

The members of TRIM protein family determine the specific recognition of target proteins, thereby regulating the ubiquitination of regulatory proteins and thus participating in a variety of intracellular physiological processes [26, 27]. An increasing number of studies demonstrate that TRIM proteins have both positive and negative regulatory roles in carcinogenesis, and that changes in the expression of some TRIM proteins are closely associated with cancer development and prognosis. In this study, we demonstrated that TRIM56 is upregulated in human glioma and functions not as a ubiquitin ligase in glioma, but as a deubiquitinating enzyme to stabilize the expression of the apoptosis inhibitor cIAP1. We found that TRIM56 promoted malignant behavior in vitro and in orthotopic xenograft models in mice that might be mediated by cIAP1. These results illuminate a role for TRIM56 as a potential oncogene in the development of human glioma and highlight its possible function as a deubiquitinating enzyme.

To date, few functions have been attributed to the TRIM family member TRIM56. The earliest studies were based on the probable antiviral and immunomodulatory functions of TRIM56 as a member of the TRIM family [28]. One study has reported that TRIM56 E3 ligase-induced monoubiquitylation of the cytosolic sensor cyclic GMP-AMP (cGAMP) synthase (cGAS) is important for cytosolic DNA sensing and IFNαβ production to induce anti-DNA virus immunity [29]. One of the earliest clues that TRIM56 might be involved in cancer was that TRIM56 is amplified in most primary effusion leukemia (PEL) cell lines and is involved in cell signaling, metabolism and protein maturation [30]. Furthermore, TRIM56 has been shown to be an oncogene in lung and breast cancers [31, 32], but a tumor suppressor in acute myeloid leukemia (AML). In our study, we found that TRIM56 expression was elevated in gliomas and associated with poor survival of glioma patients. In both in vitro assays and in vivo xenograft models in mice demonstrated that TRIM56 depletion significantly suppressed tumor progression. TRIM56 expression levels are in this context not consistent across tumor types, which may indicate that TRIM56 exerts different cancer-promoting or cancer-suppressing functions in different tumor types [32, 33].

Previous studies of TRIM56 have suggested that TRIM56 functions as a ubiquitin ligase in regulating cancer progression, due to its RING-finger domain. For example, in Kaposi's sarcoma, vFLIP was found to degrade SAP18 through the ubiquitin–proteasome pathway by recruiting the E3 ubiquitin ligase TRIM56 [34]. TRIM56 has also been shown to increase FOXM1 protein levels, enhance the stability of FOXM1 by de-ubiquitination, and promote DNA damage repair through FOXM1 in glioblastoma cells. TRIM56 may therefore suppress the radiosensitization of human glioblastoma by regulating FOXM1-mediated DNA repair [35]. However, our study demonstrated that TRIM56 can function as a deubiquitinating enzyme. Thus, TRIM56 function may be dependent on the cellular environment, and thus requires further study in diverse tumor types to more fully understand the roles of TRIM56 in the development of cancer.

The domains of the protein often provide clues as to the function of the protein. The TRIM family of proteins has a highly conserved RBCC structural domain, with a RING zinc finger structure, one or two B-box structural domains and a coiled helix structural domain in order from the N-terminus to the C-terminus, followed by a highly variable C-terminal structural domain. Most proteins containing a RING-finger domain function as an E3 ubiquitin ligase, although not all TRIM proteins containing a RING finger structural domain are E3 ubiquitin ligases [4]. In this study, we found that the zinc finger structural domain of TRIM56 could directly interact with cIAP1 causing deubiquitylation in GBM. This finding is consistent with other studies in several types of cancer showing that cIAP1 functioned as an E3 ubiquitin ligase to regulate oncogenic pathways via directly binding to key effectors of apoptosis [36, 37]. However, in addition to the currently known functions, these domains may have other unknown physiological functions. More studies are therefore required to clarify the role of a particular TRIM protein in a given tumor and the specific function of each structural domain alone or in combination with other structural domains.

**Fig. 7:**
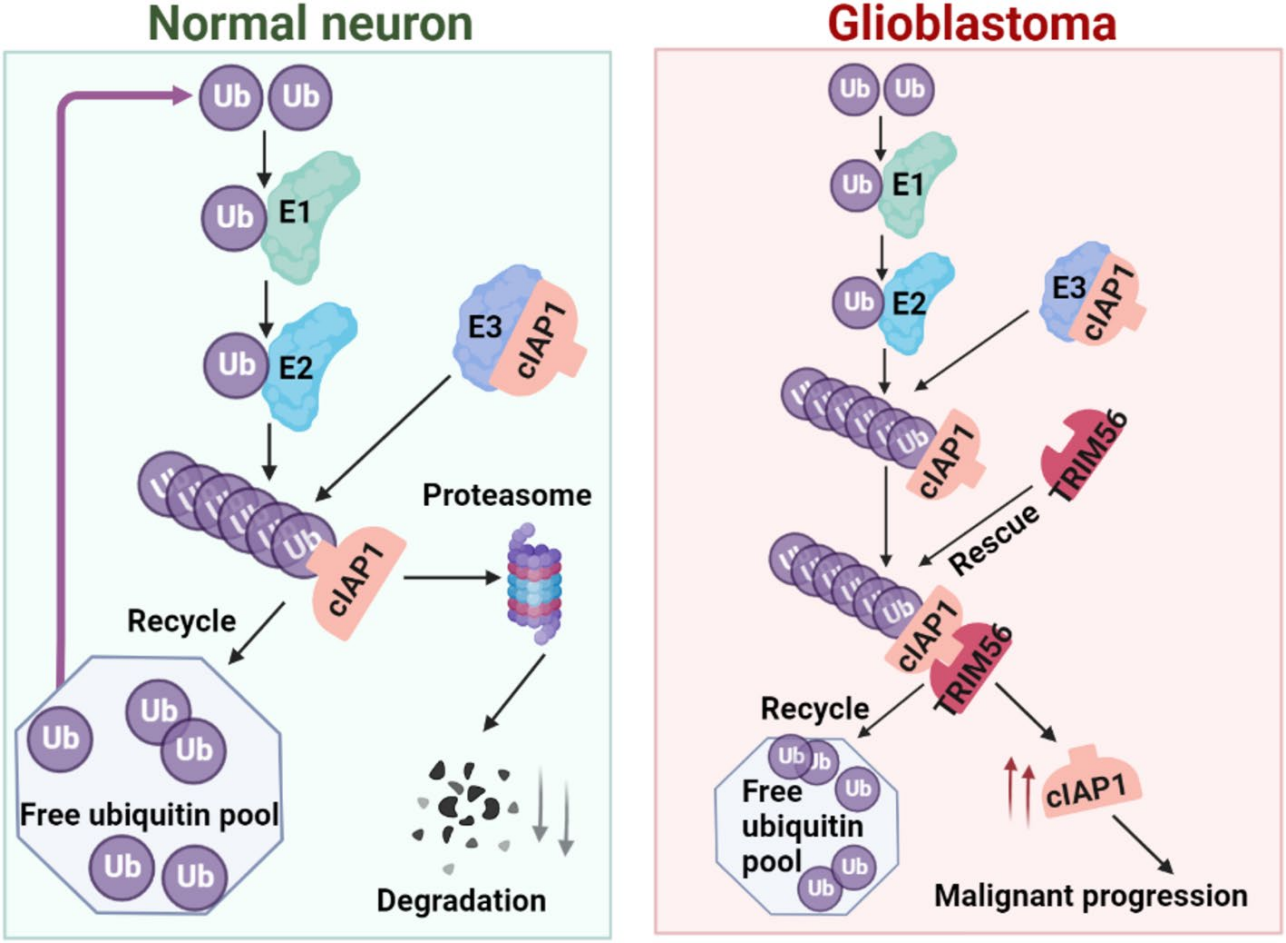
A graphic of TRIM56 inhibiting the degradation of cIAP1 and promoting glioma progression through a deubiquitylation function

In summary, we demonstrated that TRIM56 is elevated in human gliomas and knockdown of the protein inhibits tumor growth in an orthotopic GBM xenograft model in mice. TRIM56 potentially promotes glioma development as a deubiquitinating enzyme of the apoptosis inhibitor cIAP1 (Fig. 7), thereby stabilizing the protein, inhibiting glioma cell apoptosis and promoting glioma cell proliferation. Thus, TRIM56 or proteins downstream of TRIM56 may be new targets to investigate for the diagnosis and treatment of human glioma.

## Conclusions

In conclusion, our study revealed a TRIM56-cIAP1 axis that promotes malignant progression in glioma. These findings not only elucidate the underlying role of TRIM56 in driving migration and proliferation of glioma cells in vitro and in vivo, but may also provide a theoretical basis for the use of TRIM56 in the diagnosis and treatment of glioma.

## Supplementary Information

**Additional file 1:** **Figure S1.** TRIM56 is elevated in glioma and associated with poor prognosis in glioma patients. **Figure S2.** TRIM56 expression in specific molecular subtypes of gliomas in CGGA. **Figure S3.** Status/location of TRIM56 in cells within the GBM microenvironment. **Figure S4.** TRIM56 increase promotes GBM progression in vitro. **Figure S5.** TRIM56 promotes malignant progression of glioma in vivo. **Figure S6.** RNA-seq analysis reveals potential biological signaling and functions regulated by TRIM56 in glioma. **Figure S7.** cIAP1 is a downstream protein molecule of TRIM56 in glioma. **Table S1.** TRIM family members in TCGA-GBM, GSE108474 and CGGA datasets. **Table S2.** Univariate and multivariate Cox regression analysis in patients with glioma. **Table S3.** Oligonucleotide sets used in this study. **Table S4.** Plasmids used in this study. **Table S5.** Primer sets used in this study.

## Data Availability

Datasets and other files generated, analyzed, or used during this study are available from the corresponding author upon reasonable request.

## Abbreviations

TRIM: The tripartite motif
GBM: Glioblastoma multiforme
qRT-PCR: Real-time quantitative PCR
IF: Immunofluorescence
IHC: Immunohistochemistry
IP: Immunoprecipitation
Co-IP: Co-Immunoprecipitation
TNFα: Tumor necrosis factor alpha
PRRs: Pattern recognition receptors
TLR: Toll-like receptors
CNS: Central Nervous System
WB: Western blotting
CHX: Cycloheximide
LGG: Low grade glioma
TCGA: The Cancer Genome Atlas
CGGA: Chinese Glioma Genome Atlas
GSCs: GBM stem-like cells
DMEM: Dulbecco's modified Eagle's medium
GSEA: Gene set enrichment analysis
SEM: The standard error of the mean
shRNA: Short hairpin RNA
IAPs: Inhibitors of apoptosis
cIAP1: Apoptosis inhibitor 1
TNFAIP3: Tumor necrosis factor, alpha-induced protein 3
OE: Overexpression
KD: Knockdown
Ub: Ubiquitin
PEL: Primary effusion leukemia
AML: Acute myeloid leukemia
