# Supplementary material for: TRIM56 promotes malignant progression of glioblastoma by stabilizing cIAP1 protein
Source: J Exp Clin Cancer Res. 2022 Dec 6;41:336. doi: 10.1186/s13046-022-02534-8 (PMC9724401; doi:10.1186/s13046-022-02534-8)
Supplement: Supplementary file 1 — Additional file 1: Figure S1. TRIM56 is elevated in glioma and associated with poor prognosis in glioma patients. Figure S2. TRIM56 expression in specific molecular subtypes of gliomas in CGGA. Figure S3. Status/location of TRIM56 in cells within the GBM microenvironment. Figure S4. TRIM56 increase promotes GBM progression in vitro. Figure S5. TRIM56 promotes malignant progression of glioma in vivo. Figure S6. RNA-seq analysis reveals potential biological signaling and functions regulated by TRIM56 in glioma. Figure S7. cIAP1 is a downstream protein molecule of TRIM56 in glioma. Table S1. TRIM family members in TCGA-GBM, GSE108474 and CGGA datasets. Table S2. Univariate and multivariate Cox regression analysis in patients with glioma. Table S3. Oligonucleotide sets used in this study. Table S4. Plasmids used in this study. Table S5. Primer sets used in this study. [file 13046_2022_2534_MOESM1_ESM.docx]

Supplementary Information for

**TRIM56 promotes malignant progression of glioblastoma by stabilizing cIAP1 protein**

Xu Yang, Yan Zhang, Zhiwei Xue, Yaotian Hu, Wenjing Zhou, Zhiyi Xue, Xuemeng Liu, Guowei Liu, Wenjie Li, Xiaofei Liu, Xingang Li, Mingzhi Han * and Jian Wang*

*These authors contributed equally to this work as senior authors.

Email: Dr. Mingzhi Han: [mingzhi.han@sdu.edu.cn](mailto:mingzhi.han@sdu.edu.cn); Dr. Jian Wang: [jian.wang@uib.no](mailto:jian.wang@uib.no).

**Contents**

[Supplementary Figures 3](#_Toc93434579)

[Supplementary Tables 15](#_Toc93434580)


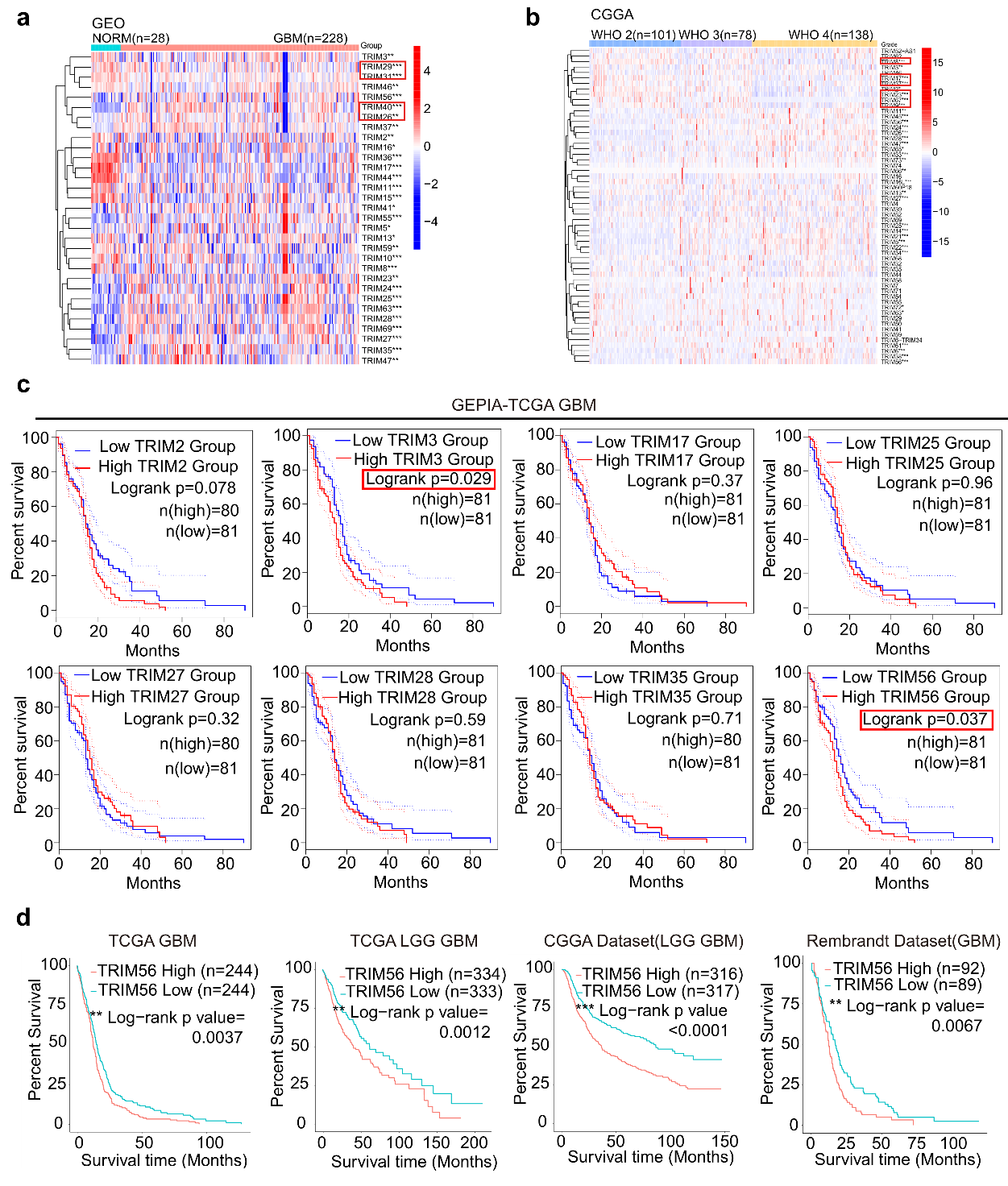


**Figure S1. TRIM56 is elevated in glioma and associated with poor prognosis in glioma patients.**

**(**a**)** Heat map of differentially expressed TRIM family genes between normal brain tissue (n = 28) and glioblastoma (n = 228) from the GEO dataset at [GSE108](https://www.ncbi.nlm.nih.gov/geo/query/acc.cgi?acc=GSE14468)474. Gene expression values are z-transformed. The four genes with the most pronounced variability are highlighted in the red boxes.

**(**b**)** Heat map of differentially expressed TRIM family genes between WHO grade 2 (n = 101), grade 3 (n = 78) and grade 4 (n = 138) gliomas from the CGGA dataset. Gene expression values are z-transformed. The three genes with the most pronounced variability are highlighted in the red boxes.

**(**c**)** Kaplan-Meier survival curves of patients with high and low expression of *TRIM56* based on the TCGA-GBM dataset. Patients were designated as high or low expression based on the median as the intercept value. The log rank test was used to determine the significance of the difference.

**(**d**)** Kaplan-Meier survival curves of patients with high and low expression of *TRIM56* based on the TCGA-GBM, TCGA-LGG GBM, CGGA and Rembrandt datasets. Patients were designated as high or low expression based on the median as the intercept value. The log rank test was used to determine the significance of the difference. *p < 0.05; **p < 0.01; ***p < 0.001; ****p < 0.0001.

**
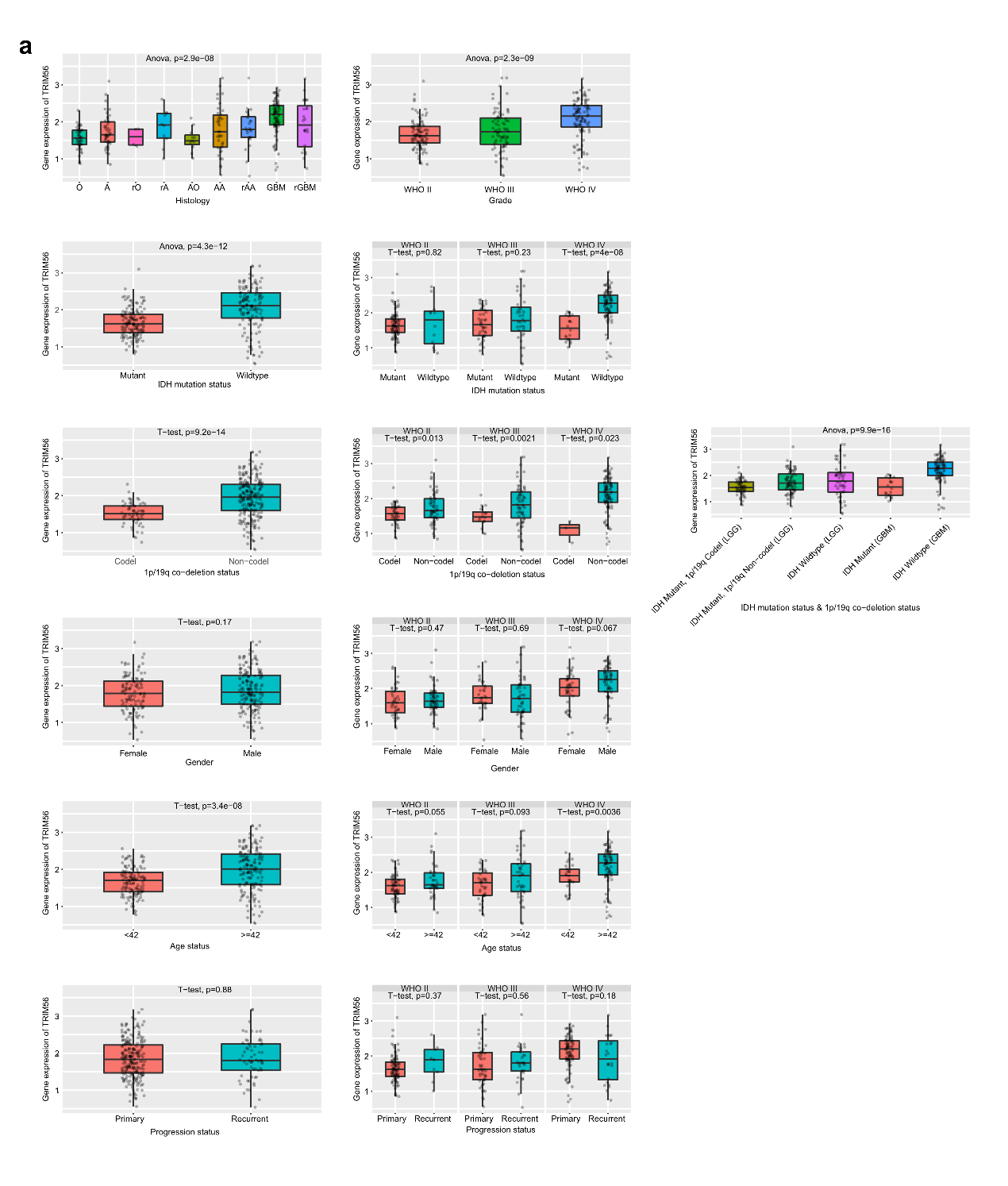
**

**Figure S2. TRIM56 expression in specific molecular subtypes of gliomas in CGGA.**

**
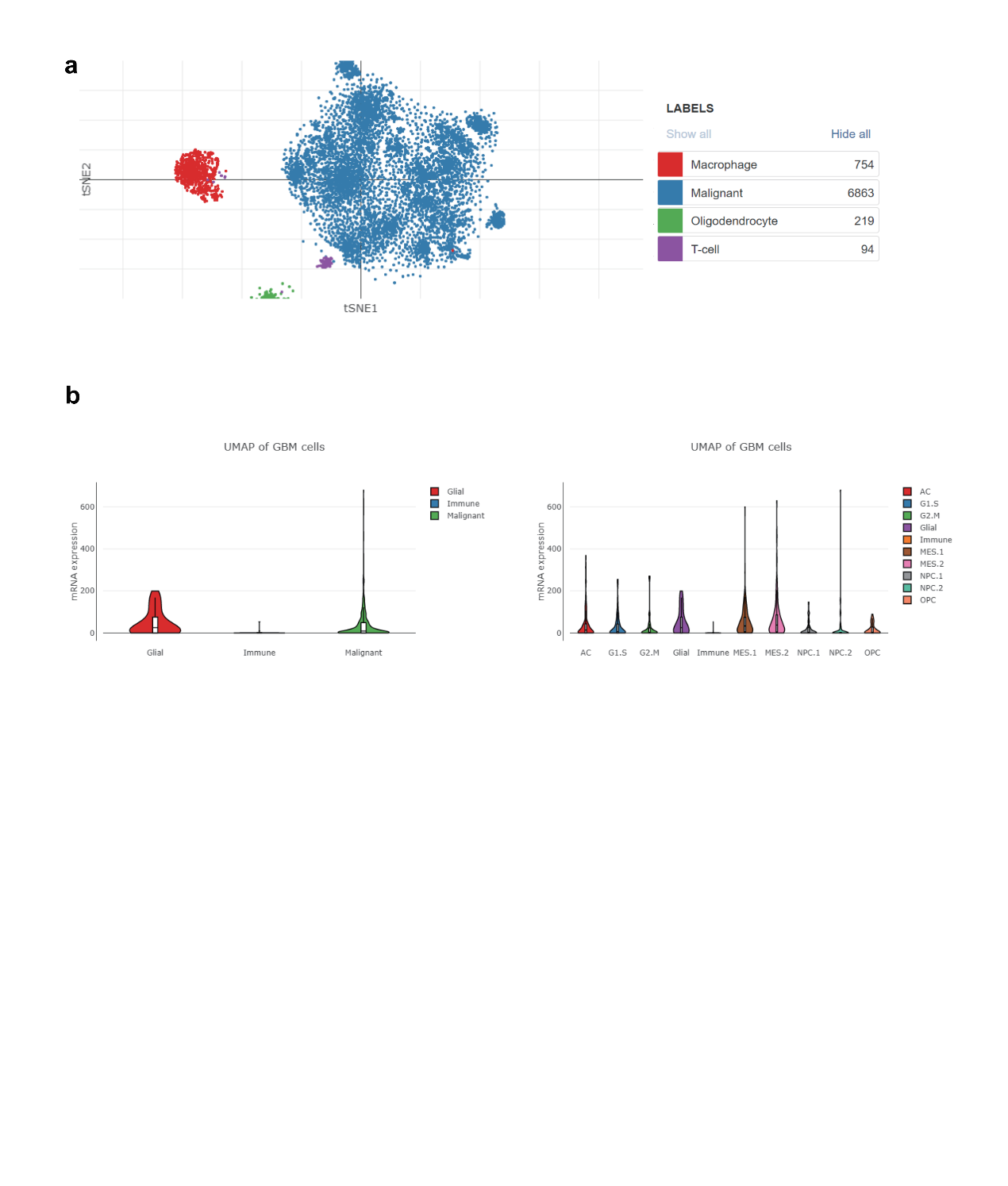
**

**Figure S3. Status/location of TRIM56 in cells within the GBM microenvironment.**

**(**a**)** TRIM56 status/location in IDH-wt GBM tSNE (all cells) from the website Single Cell Portal. Red represents macrophages; blue, malignant cells; green, oligodendrocytes; and purple, T cells.

**(**b**)** The statistical graph of TRIM56 expression in IDH-wt GBM UMAP (all cells) from the website Single Cell Portal. The legend to the right of the statistical chart indicates the cell types represented by the different colors.

**
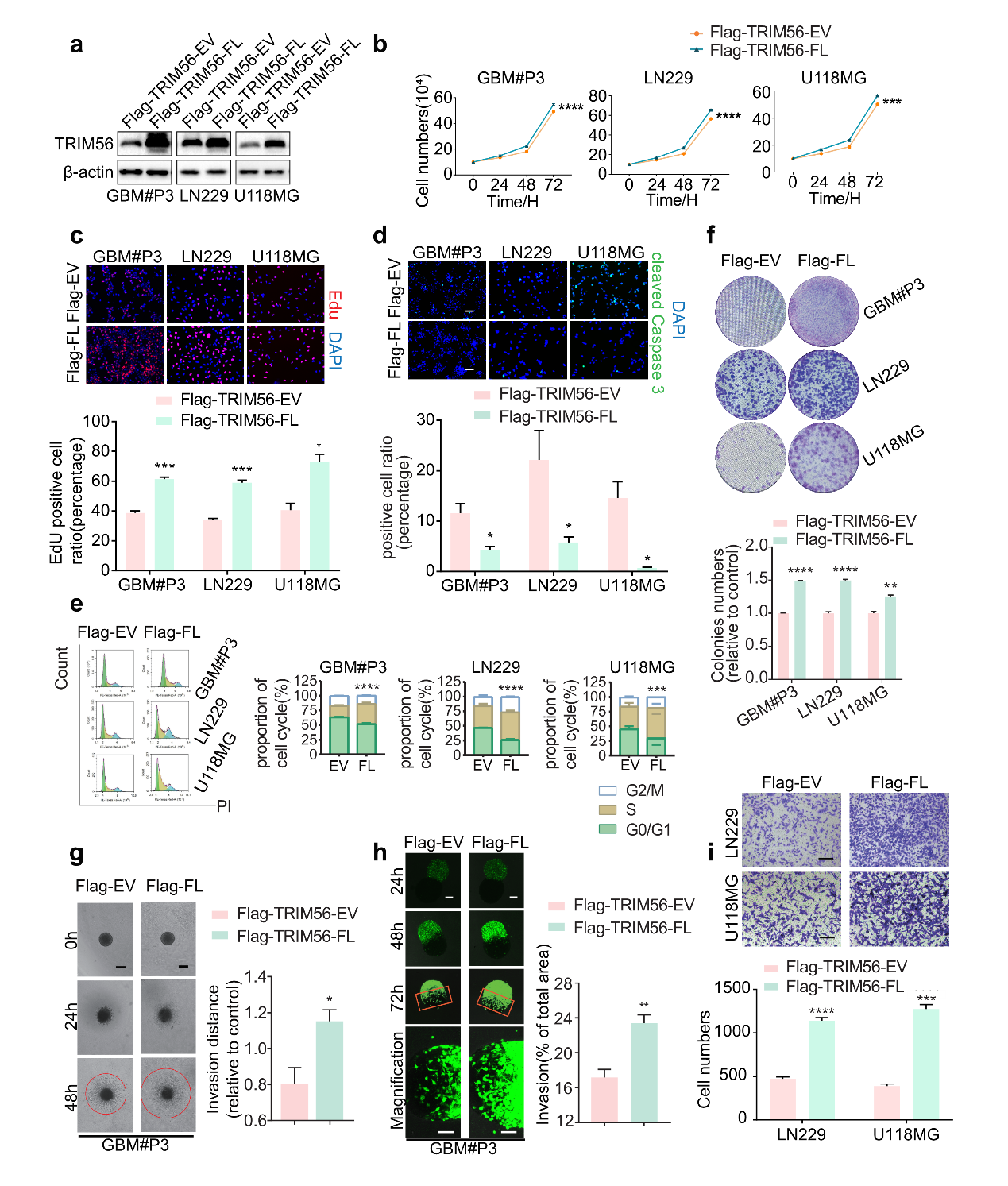
**

**Figure S4. TRIM56 increase promotes GBM progression in vitro.**

**(**a**)** Western blot showing TRIM56 protein levels in GBM#P3, LN229 and U118MG cells transfected with the indicated plasmid, Flag-TRIM56-EV (empty vector) or Flag-TRIM56-FL (full length).

**(**b**)** Growth curves generated from cell number counts for the indicated cells performed over a 72-hour period (at 24-hour intervals).

**(**c**)** Fluorescence images and quantification of EdU assays to assess proliferation of the indicated cells (scale bar, 500 μm).

**(**d**)** Fluorescence images and quantification of immunofluorescence staining of cleaved caspase-3 in the indicated cells (scale bar, 500 μm). Nuclei were stained with DAPI.

**(**e**)** Cell cycle analysis for the indicated cells. The percentage of cells arrested in the G0/G1 phase is analyzed in the bar graphs (right panels).

**(**f**)** Representative images of colony formation assays and quantification of the indicated cells, seeded at 500 cells/well and cultured for 2 weeks. Cells were fixed, stained with crystal violet and counted.

**(**g**)** Representative images of 3D tumor sphere invasion assays and quantification for GBM#P3 cells indicated in (a). Representative images at 0 h, 24 h and 48 h are shown, and quantitative plots show the percentage of invaded area (scale bar, 200 μm).

**(**h**)** Representative images of co-culture invasion assays and quantification of GBM#P3 cells indicated in (a). Invasion was assessed at 24 h, 48 h and 72 h. Scale bars = 200 μm (magnified insets).

**(**i**)** Representative images of Transwell assays and quantification to assess the migration and invasive ability of LN229 U118MG cells indicated in (a) (scale bar, 50 μm).

Comparisons between two independent samples were performed using two-tailed t tests or two-way ANOVA. Error bars indicate at least three independent experiments, and data are shown as mean ± SEM. ∗p < 0.05, ∗∗p < 0.01, ∗∗∗p < 0.001, and ∗∗∗∗p < 0.0001.


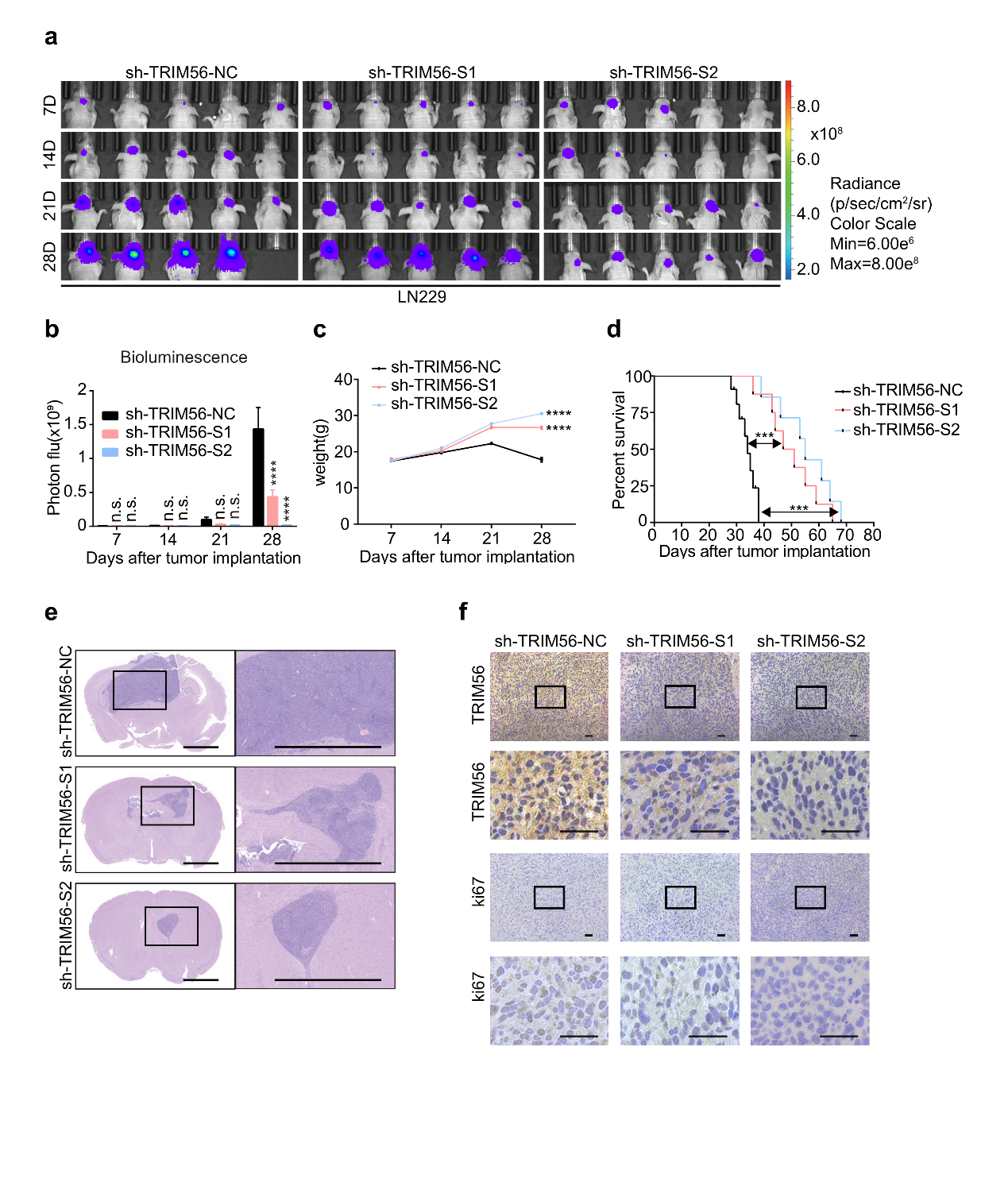


**Figure S5. TRIM56 promotes malignant progression of glioma in vivo.**

**(**a-b**)** In vivo bioluminescence imaging and quantification of orthotopic xenografts derived from LN229 sh-TRIM56-NC, -S1, and -S2 cells in nude mice at the indicated time points (data from 5 animals/group). Representative bioluminescence images at day 7, day 14, day 21 and day 28 post-implantation are shown. Two-way ANOVA and log rank analysis were used to determine the statistical significance of bioluminescence on day 28 (P1 < 0.01; P2 < 0.001). Data are shown as the mean ± SEM.

**(**c-d**)** Weight and Kaplan-Meier analysis of survival time for nude mice implanted with LN229-sh-TRIM56-NC, -S1 and -S2 cells performed at the indicated time points. Data are shown as the mean ± SEM of three independent experiments. Two-way ANOVA and log-rank analysis were used to determine statistical significance (scale bar, 100 µm).

**(**e**)** Representative images of HE staining of brain sections from xenografted mice on the day of euthanasia, showing representative macroscopic images. Scale bar = 2 mm.

**(**f**)** Representative images of immunostaining of TRIM56 and Ki67 in xenograft sections (scale bar, 100 µm).

Comparisons between two independent samples and among multiple samples were performed using two-tailed t tests and one/two-way ANOVA, respectively. Error bars indicate at least three independent experiments, and data are shown as mean ± SEM. ∗p < 0.05, ∗∗p < 0.01, ∗∗∗p < 0.001, and ∗∗∗∗p < 0.0001.


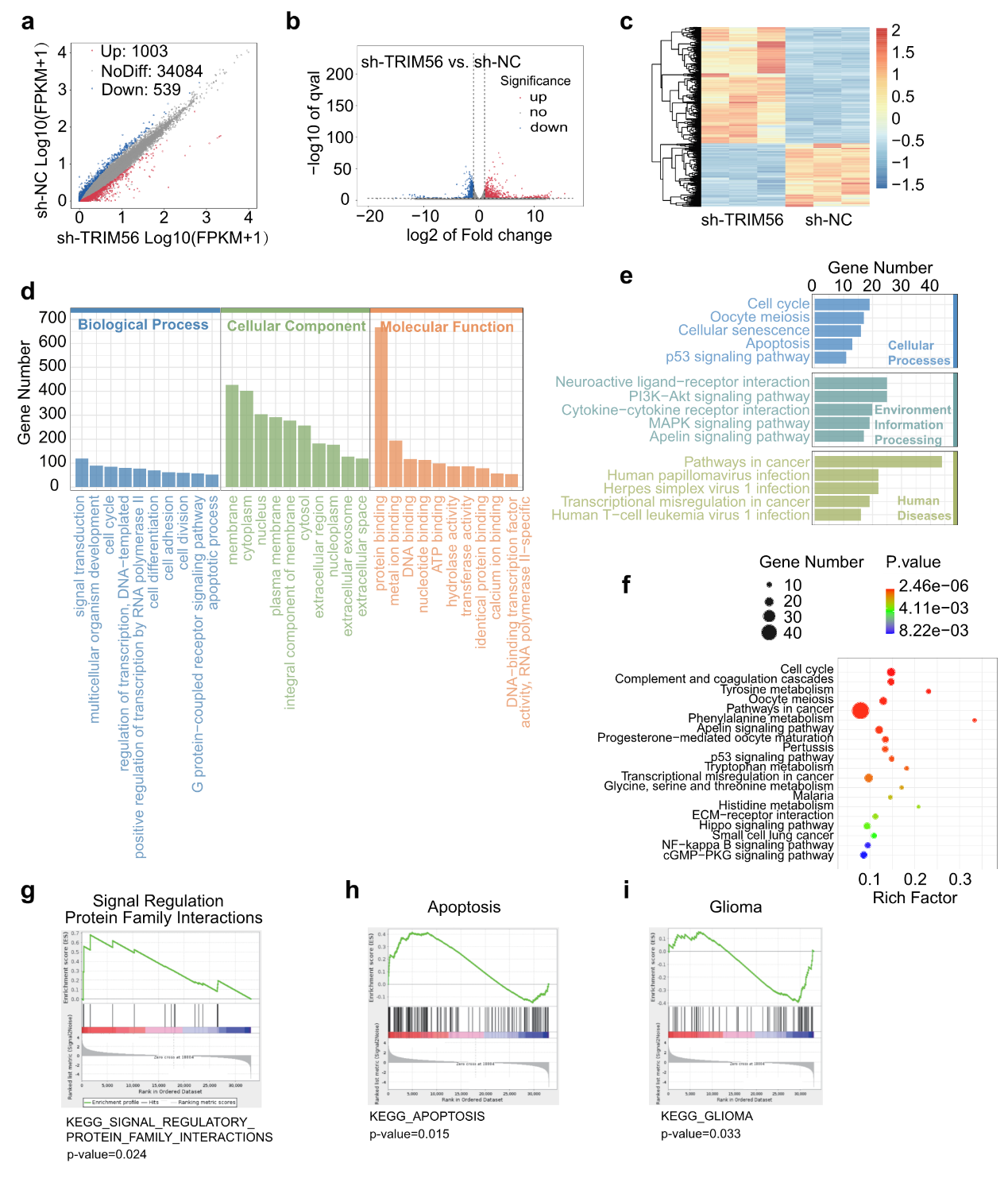


**Figure S6. RNA-seq analysis reveals potential biological signaling and functions regulated by TRIM56 in glioma.**

**(**a**)** Scatterplot of differentially expressed genes in LN229-sh-TRIM56 cells vs -sh-NC cells. The values on the X and Y axes of the scatter plot values are the average FPKM values for each group (on a scale of 10). Points above the top line (1003 red dots) or below the bottom line (539 blue dots) indicate genes with more than 2-fold change in expression between the two groups.

**(**b**)** Volcano plots of differentially expressed genes. The values on the X and Y axes of the volcano plot are the fold change (log-transformed) values and q values (-log10 transformed) between the two groups, respectively. Red/blue dots indicate differential genes with statistically greater than 2-fold change in expression.

**(**c**)** Heat map of differentially expressed transcripts in RNA-seq data from LN229-sh-TRIM56 and sh-NC (control). Gene expression data are mean z-transformed to show that high expression is in red and low expression is in blue.

**(**d-f**)** Gene Ontology (GO) and Kyoto Encyclopedia of Genes and Genomes (KEGG) analyses revealed the potential roles of differentially expressed genes following knockdown of TRIM56 in LN229 cells.

**(**g-i**)** Representative GSEA results show that apoptosis and signal regulation protein family interaction are closely correlated with *TRIM56* expression.

*p < 0.05; **p < 0.01; ***p < 0.001; ****p <0.0001.


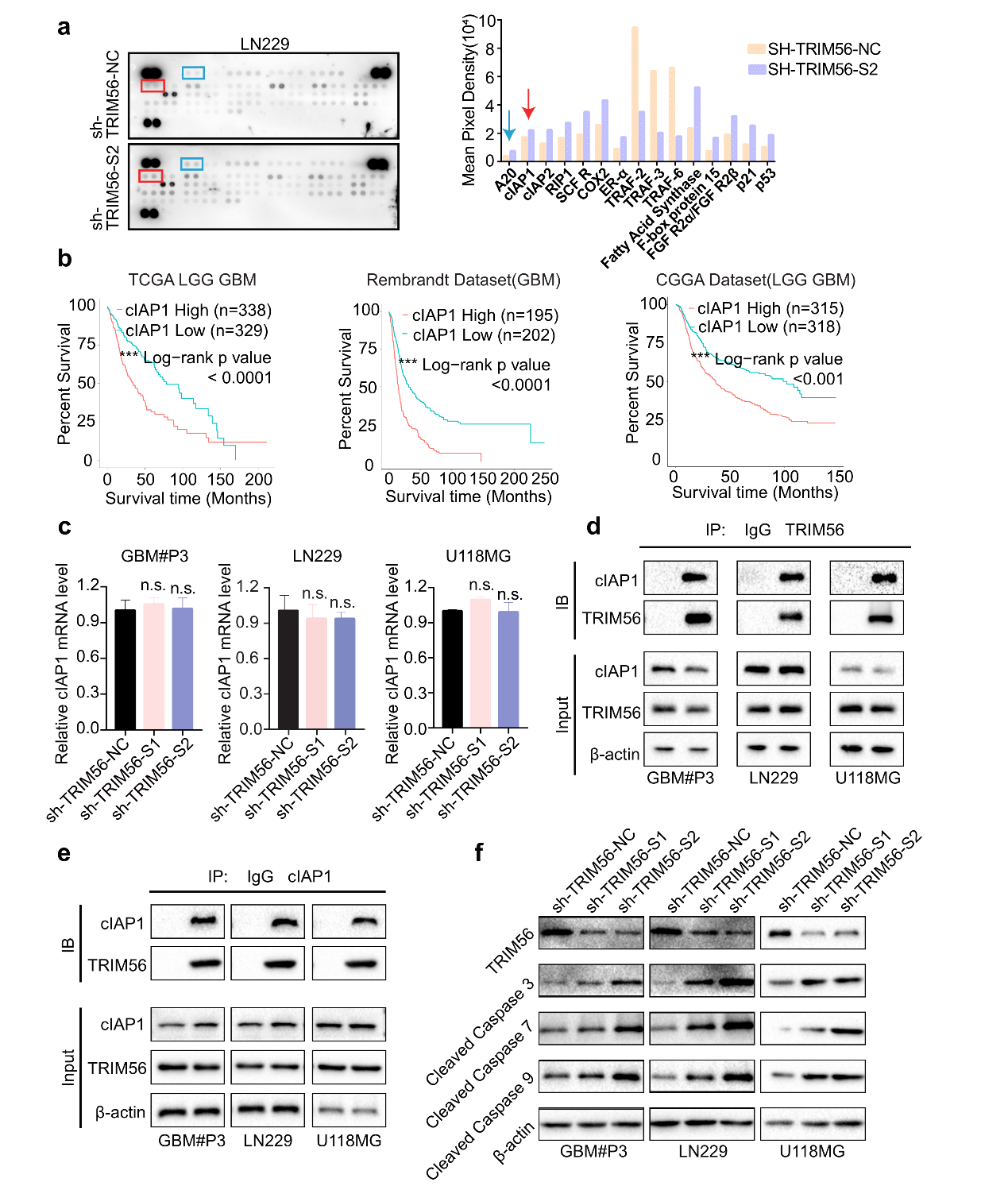


**Figure S7. cIAP1 is a downstream protein molecule of TRIM56 in glioma.**

(a) Images of Human Ubiquitin Array (500 μg lysate, 2 min exposure) performed with the lysates prepared from LN229-sh-NC and -sh-TRIM56 cells treated with 20 μg/mL of the proteasome inhibitor MG132 for 6 h before collection. Specific quantification of spots with significantly elevated levels of ubiquitination was performed and the two candidate spots are highlighted in the colored boxes.

(b) Kaplan-Meier survival curves for patients with high and low *TRIM56* expression based on TCGA-GBM, TCGA-LGG GBM, CGGA and Rembrandt datasets. Patients were designated as high or low expression based on the median as the intercept value. The log-rank test was used to determine the significance of differences.

(c) qRT-PCR analysis performed to detect the relative mRNA expression of *cIAP1* in GBM#P3-, LN229- and U118MG-sh-TRIM56-NC, -S1 and -S2.

(d-e) Western blot of immunoprecipitations performed on GBM#P3, LN229 and U118MG cells with IgG or TRIM56/cIAP1 antibodies. Blots were incubated with antibodies against TRIM56 or cIAP1, IgG and β-actin.

(f) Western blot of the levels of cIAP1-related proteins (cleaved caspase-3, cleaved caspase-7, and cleaved caspase-9) in the indicated cells.

Comparisons between two independent samples and among multiple samples were performed using two-tailed t tests and one/two-way ANOVA, respectively. Error bars indicate at least three independent experiments, and data are shown as mean ± SEM. ∗p < 0.05, ∗∗p < 0.01, ∗∗∗p < 0.001, and ∗∗∗∗p < 0.0001.

**Supplementary Table S1.** **TRIM family members in TCGA-GBM, GSE108474 and CGGA datasets**

**Supplementary Table S2.**

**
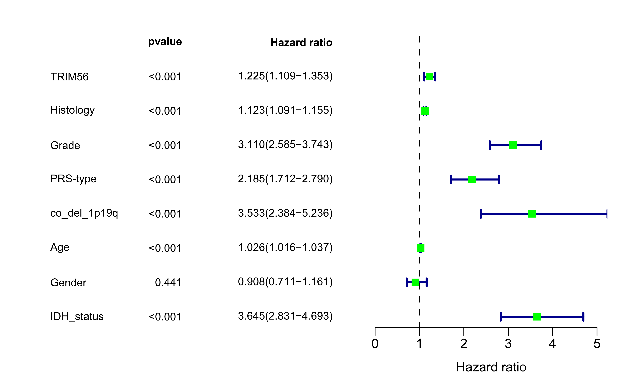

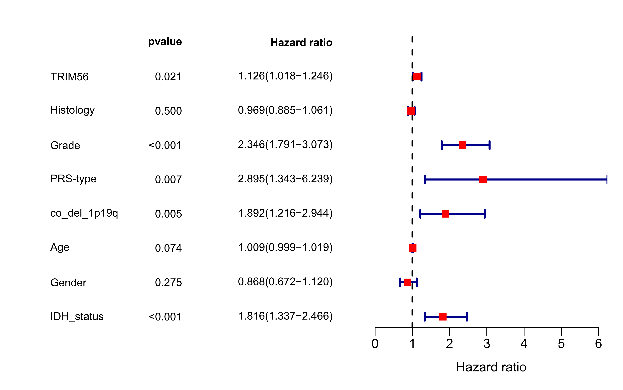
Univariate and multivariate Cox regression analysis in patients with glioma**

1. **
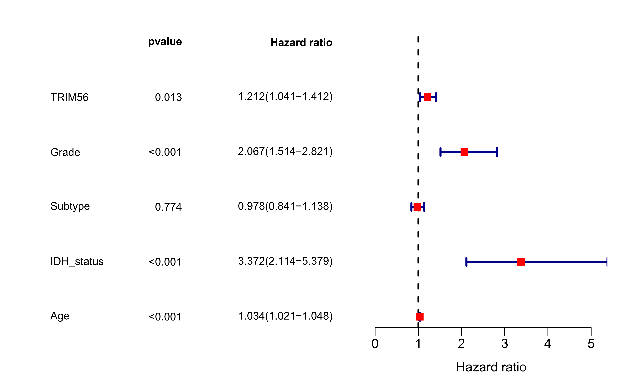

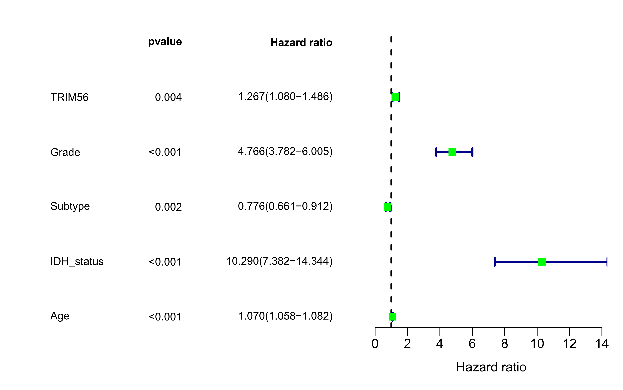
**Univariate and multivariate Cox regression analysis in CGGA
2. Univariate and multivariate Cox regression analysis in TCGA

**Supplementary Table S3. Oligonucleotide sets used in this study**

| **si/shRNAs** | **Sequences** |
| --- | --- |
| si-NC | 5'-UUCUCCGAACGUGUCACGUTT-3' |
| si-*TRIM56-1* | 5'-CCACGTGGAGGTGTACAAT-3' |
| si-*TRIM56-2* | 5'-GGAGGTGTACAATATGGAA-3' |
| sh-NC  sh-TRIM56-1  sh-TRIM56-2 | 5'-TTCTCCGAACGTGTCACGT-3'  5'-CCACGTGGAGGTGTACAAT-3'  5'-GGAGGTGTACAATATGGAA-3' |

**Supplementary** **Table S4. Plasmids used in this study**

| **Plasmids** |  | **Resources** |
| --- | --- | --- |
| pcDNA3.1-Flag-empty vector |  | Biosune Biotechnology |
| pcDNA3.1-Flag-TRIM56-full length |  | Biosune Biotechnology |
| pcDNA3.1-Flag-TRIM56-△ZF |  | Biosune Biotechnology |
| pcDNA3.1-Flag-TRIM56-△CC |  | Biosune Biotechnology |
| pcDNA3.1-Flag-TRIM56-△M |  | Biosune Biotechnology |
| pcDNA3.1-MYC-empty vector |  | Biosune Biotechnology |
| pcDNA3.1-MYC- cIAP1 |  | Biosune Biotechnology |
| pcDNA3.1-His- Ub |  | Biosune Biotechnology |

**Supplementary** **Table S5. Primer sets used in this study**

| **Primer set** | **Primers** | **Sequence (5’-3’)** | **Product size (bp)** |
| --- | --- | --- | --- |
| ***cIAP1***  ***GAPDH*** | F  R  F  R | 5'- TCCAGCCTTTCTCCAAACCC -3'  5'- ACCAGCTCTTGCCAATTCTGA -3'  5’-GCACCGTCAAGGCTGAGAAC-3’  5’-TGGTGAAGACGCCAGTGGA-3’ | **165**  **138** |
